# Prospective Associations of Body Composition and Body Shape With the Risk of Developing Pancreatic Cancer in the UK Biobank Cohort

**DOI:** 10.1002/cam4.70809

**Published:** 2025-03-25

**Authors:** Sofia Christakoudi, Konstantinos K. Tsilidis, Marc J. Gunter, Elio Riboli

**Affiliations:** ^1^ Department of Epidemiology and Biostatistics, School of Public Health Imperial College London London UK; ^2^ Department of Hygiene and Epidemiology University of Ioannina School of Medicine Ioannina Greece

**Keywords:** cancer prevention, cancer risk factors, epidemiology, pancreatic cancer

## Abstract

**Background:**

Obesity and diabetes are positively associated with pancreatic cancer risk. It is unclear, however, whether fat or fat‐free mass plays a role in these relationships, whether abdominal obesity is more important than general obesity or whether the associations with anthropometric indices and diabetes are independent of each other.

**Methods:**

We used multivariable Cox proportional hazards models to examine the prospective associations of body composition (allometric fat‐mass index (AFI) and allometric lean‐mass index (ALI), based on bioelectrical impedance, uncorrelated with each other and with height), waist size (allometric waist‐to‐hip index (WHI), uncorrelated with weight and height) and diabetes with pancreatic cancer risk in UK Biobank. We tested heterogeneity by sex, age and follow‐up time with the augmentation method (p_het).

**Results:**

During a mean follow‐up of 10.4 years, 999 pancreatic cancer cases were ascertained in 427,939 participants. AFI was positively associated with pancreatic cancer risk in participants overall, independent of ALI, WHI, diabetes and covariates (hazard ratio HR = 1.102; 95% confidence interval CI = 1.033–1.176 per 1 standard deviation (SD) increase), more strongly in women aged under 55 years at recruitment (HR = 1.457; 95% CI = 1.181–1.797; p_het = 0.007) and in men only for follow‐up 6 years or longer (HR = 1.159; 95% CI = 1.037–1.295; p_het = 0.075). ALI was positively associated with pancreatic cancer risk in participants overall (HR = 1.072; 95% CI = 1.005–1.145), more specifically in men (HR = 1.132; 95% CI = 1.035–1.238; p_het = 0.091). A positive association of WHI with pancreatic cancer risk was observed only in unadjusted models but was lost after adjustment for smoking status and diabetes. Independent of anthropometric indices, diabetes was associated positively with pancreatic cancer risk in participants overall (HR = 1.688; 95% CI = 1.365–2.087), but in women only for follow‐up under 6 years (HR = 2.467; 95% CI = 1.477–4.121; p_het = 0.042).

**Conclusions:**

General obesity (reflected in AFI and ALI) and diabetes but not abdominal obesity were associated positively with pancreatic cancer risk, independent of each other and covariates.

## Graphical abstract

Using bioelectrical impedance measurements from the UK Biobank, we have shown that the allometric fat‐mass and lean‐mass indices (reflecting general obesity), as well as diabetes, were associated positively with pancreatic cancer risk, independent of each other and other known risk factors. No independent association of abdominal obesity with pancreatic cancer risk remained after factoring out the associations of waist size with general obesity and height and accounting for smoking and diabetes.

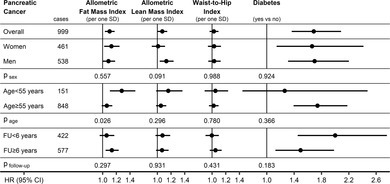

**Abbreviations**
ABSI: a body shape index
AFI: allometric fat‐mass index
ALI: allometric lean‐mass index
AVI: abdominal volume index
BMI: body mass index
BRI: body roundness index
CI: confidence interval
ConI: conicity index
FFM: fat‐free mass
FM: fat mass
HC: hip circumference
HI: hip index
HR: hazard ratio
ICD10: 10th revision of the International Statistical Classification of Diseases
SD: standard deviation
UK: United Kingdom
WC: waist circumference
WHI: waist‐to‐hip index
WHR: waist‐to‐hip ratio
WHRadjBMI: waist‐to‐hip ratio adjusted for BMI
WHtR: waist‐to‐height ratio
WWI: weight‐adjusted waist index

## 1 Introduction

Pancreatic cancer is ranked 14th according to incidence, with almost half a million new cases worldwide in 2020 (2.6% of all incident cancer cases) but, with a similar number of deaths, it reaches 7th for cancer mortality (4.7% of all cancer‐related deaths) [1] and has as low as 8% five‐year survival in Europe [2]. It is therefore important to understand the relevant modifiable risk factors to facilitate the prevention of this malignancy. Notably, pancreatic cancer is one of the 13 obesity‐related cancers [3] and the proportion of disability‐adjusted life years due to pancreatic cancer that is attributable to high body mass index (BMI) has increased over the last three decades [4]. Pancreatic cancer also has a complex two‐way relationship with type 2 diabetes, in which long‐standing diabetes can present as a risk factor while new‐onset diabetes can be induced by the developing pancreatic tumour [5]. Considering that abdominal rather than general obesity is more specifically associated with diabetes [6], a question emerges as to whether the association of obesity with pancreatic cancer risk is confined to general obesity reflected in BMI or is determined mainly by abdominal obesity.

Observational studies using waist circumference and waist‐to‐hip ratio (WHR) as waist indices have shown that abdominal size, as well as BMI, is positively associated with pancreatic cancer risk [7]. Traditional waist indices, however, are correlated positively with BMI (strongly for waist circumference and moderately for WHR) and thus reflect general as well as abdominal obesity [8]. A study examining associations with WHRadjBMI (derived as the residuals of WHR regressed on BMI and uncorrelated with BMI) in a Mendelian randomisation (MR) framework concluded that abdominal but not general obesity is more important for pancreatic cancer risk [9]. Partly in agreement, examining the waist‐to‐hip index (WHI), which is the allometric analogue of the WHR and is uncorrelated with BMI, we have previously shown a borderline positive association of WHI with pancreatic cancer risk in women from the UK Biobank [8]. In this same study, however, we did not adjust for diabetes, but we also showed a stronger positive association of BMI with pancreatic cancer risk in premenopausal women. At the same time, a different study in the UK Biobank, examining separately men and women and adjusting fat mass (FM) and fat‐free mass (FFM) measured with bioelectrical impedance (BIA) for each other and height, failed to find any material evidence for an association of body composition with pancreatic cancer risk [10]. FM and FFM, however, are both related to obesity, as both increase in studies of overfeeding [11] and, correspondingly, are substantially positively correlated with each other [12]. They would also be higher in taller individuals, and height is associated positively with pancreatic cancer risk [13]. Therefore, mutually adjusting FM, FFM and height in the same model is likely to produce biased risk estimates, similar to the bias arising from mutual adjustment of other highly correlated anthropometric measures—waist and hip circumferences and BMI [8].

In this study, we have used UK Biobank data, with a further follow‐up and almost twice as many incident pancreatic cancer cases compared to the previous studies [8, 10], to evaluate the independent associations of body composition, abdominal obesity and diabetes with pancreatic cancer risk in participants overall and according to sex, age and follow‐up time. We have also shown the bias in risk estimates arising from mutual adjustment of FM, FFM and height in a joint model and have compared risk estimates for a range of traditional and alternative waist indices based on geometric and allometric considerations.

## 2 Methods

### 2.1 Study Population

UK Biobank is a large prospective cohort. Half a million participants (registered with the National Health Service and living within 40 km of an assessment centre in England, Scotland and Wales) were recruited between 2006 and 2010 when aged 40–70 years [14]. We restricted this study to participants with self‐reported white ancestry, due to the limited number of participants with other ethnic backgrounds. We excluded participants with missing anthropometric measurements, a mismatch between the genetic and self‐reported sex, women pregnant at recruitment, participants with prevalent cancer at recruitment or with missing body composition measurements (Table S1). In total, we excluded 74,233 participants (14.8% from the total dataset), from whom participants had already been excluded for withdrawing consent by the time of analysis.

### 2.2 Ascertainment of Pancreatic Cancer

UK Biobank is linked to the national cancer registry of the United Kingdom. The outcome of interest in this study was the first primary pancreatic cancer diagnosed after recruitment, as defined in [8]—Code C25 from the 10th version of the International Statistical Classification of Diseases (ICD10), with behavioural codes 3 (malignant, primary site) or 5 (malignant, microinvasive), but excluding rare morphologies (histological codes 8150, 8151, 8152, 8246 and 9591). Follow‐up was censored at the date of diagnosis for first cancer in a location other than the pancreas and for first cancer in the pancreas with behavioural codes 6 (malignant, metastatic site), 9 (malignant, uncertain whether primary or metastatic site) or missing. For participants remaining free of cancer, follow‐up was censored at the earliest of the date of death or the date of the last complete cancer registry (31 March 2020 for England and Scotland and 31 December 2016 for Wales).

### 2.3 Anthropometric Indices

BIA measurements of total FM and total FFM were obtained with the Tanita BC‐418MA Body Fat Analyser (Tanita Corp, Tokyo, Japan). Height, weight, waist circumference (measured at the natural indent or the umbilicus) and hip circumference (measured at the widest point) were obtained by trained UK Biobank technicians according to established protocols [15]. BMI, calculated as weight (kg) divided by height (m) squared, was provided by UK Biobank.

As indices of body composition, we used the allometric fat‐mass index (AFI) and the allometric lean‐mass index (ALI), calculated with the power coefficients that we had previously derived in UK Biobank (separately for women and men) [16]:

$${\mathrm{AFI}}_{\text{women}}=\mathrm{FM}\;\left(\mathrm{kg}\right)*\text{Height}\;{\left(m\right)}^{-1.3582}$$

$${\mathrm{AFI}}_{\mathrm{men}}=\mathrm{FM}\;\left(\mathrm{kg}\right)*\text{Height}\;{\left(m\right)}^{-1.0290}$$

$${\mathrm{ALI}}_{\text{women}}=\mathrm{FFM}\;\left(\mathrm{kg}\right)*\text{Height}\;{\left(m\right)}^{-1.1960}*\mathrm{FM}\;{\left(\mathrm{kg}\right)}^{-0.1670}$$

$${\mathrm{ALI}}_{\mathrm{men}}=\mathrm{FFM}\;\left(\mathrm{kg}\right)*\text{Height}\;{\left(m\right)}^{-1.8122}*\mathrm{FM}\;{\left(\mathrm{kg}\right)}^{-0.1481}$$

We compared AFI and ALI with two traditionally used body composition pairs—total FM and total FFM and FM index and FFM index [10]. The latter indices are calculated by dividing FM and FFM by height (m) squared and assume that the relationship of height with the individual body composition components in both sexes is the same as the relationship of height with weight in BMI. In contrast, AFI and ALI use tissue‐specific and sex‐specific scaling coefficients for height, and ALI additionally factors out the influence of obesity on FFM, as we have previously explained [16]. To illustrate that the allometric body composition indices are effectively equivalent to body composition residuals, we derived specifically for this study (separately for women and men) residual FM (regressing total FM (kg) on height (cm)) and residual FFM [regressing total FFM (kg) on height (cm) and total FM (kg)] (Table S2).

As indices of waist size, we compared waist circumference (WC) with a range of previously derived and previously used alternative waist indices: abdominal volume index (AVI) [17]; waist‐to‐height ratio (WHtR); body roundness index (BRI) [18]; WHR; conicity index [19]; weight‐adjusted waist index (WWI) [20]; and two allometric indices—WHI [8] and allometric body shape index (ABSI) [21]. For this study, we are additionally showing transformations of the equations to illustrate the relationship of waist indices with BMI and height or with each other (for details on the transformations see Supplementary Methods). In the equations below, BMI is always measured in (kg/m^2^) and weight in (kg), but note that the units of measurement of WC, hip circumference (HC) and height differ:

$$\mathrm{AVI}=\frac{2*\mathrm{WC}{\left(\mathrm{cm}\right)}^{2}+0.7*{\left(\mathrm{WC}\left(\mathrm{cm}\right)-\mathrm{HC}\left(\mathrm{cm}\right)\right)}^{2}}{1000}$$

$$\text{WHtR}=\frac{\mathrm{WC}\left(m\right)}{\text{Height}\left(m\right)}$$

$$\mathrm{BRI}=364.2-365.5*\sqrt{1-\frac{{\left(\frac{0.5*\mathrm{WC}\left(m\right)}{\pi }\right)}^{2}}{{\left(0.5*\text{Height}\left(m\right)\right)}^{2}}}=364.2-365.5*\sqrt{1-\frac{{\text{WHtR}}^{2}}{\pi^{2}}}$$

$$\mathrm{WHR}=\frac{\mathrm{WC}\left(\mathrm{cm}\right)}{\mathrm{HC}\left(\mathrm{cm}\right)}$$

$$\text{ConI}=\frac{\mathrm{WC}\left(m\right)}{0.109*\sqrt{\frac{\text{Weight}}{\text{Height}\left(m\right)}}}=9.174*\mathrm{WC}\left(m\right)*{\mathrm{BMI}}^{-\frac{1}{2}}*\text{Height}{\left(m\right)}^{-\frac{1}{2}}$$

$$\mathrm{WWI}=\mathrm{WC}\left(\mathrm{cm}\right)*{\text{Weight}}^{-\frac{1}{2}}=100*\mathrm{WC}\left(m\right)*{\mathrm{BMI}}^{-\frac{1}{2}}*\text{Height}{\left(m\right)}^{-1}$$

$$\mathrm{WHI}=\mathrm{WHR}*{\text{Weight}}^{-\frac{1}{4}}*\text{Height}{\left(\mathrm{cm}\right)}^{\frac{1}{2}}=10*\mathrm{WHR}*{\mathrm{BMI}}^{-\frac{1}{4}}$$

$$\text{ABSI}=1000*\mathrm{WC}\left(m\right)*{\text{Weight}}^{-\frac{2}{3}}*\text{Height}{\left(m\right)}^{\frac{5}{6}}=1000*\mathrm{WC}\left(m\right)*{\mathrm{BMI}}^{-\frac{2}{3}}*\text{Height}{\left(m\right)}^{-\frac{1}{2}}$$

The transformed equations above show that BRI is a mathematical transformation of WHtR that does not involve additional variables. ConI and ABSI have the same adjustment for height, but ConI is insufficiently adjusted for BMI (the power coefficient for BMI has smaller absolute value in the equation for ConI compared to the equation for ABSI). WWI is insufficiently adjusted for BMI (similarly to ConI) but (unlike ConI) is overadjusted for height (the power coefficient for height has larger absolute value in the equation for WWI compared to the equation for ABSI). The power coefficients for weight and height in WHI correspond exactly to their proportion in BMI, hence no additional term for height is required.

Lastly, we calculated the hip index (HI) using the original coefficients [22] for women, but we used the coefficients that we had previously derived for UK Biobank men to avoid the inverse correlation of HI with BMI observed in men when using the original coefficients [8]:

$${\mathrm{HI}}_{\text{women}}=\mathrm{HC}\left(\mathrm{cm}\right)*\text{Weight}{\left(\mathrm{kg}\right)}^{-0.482}*\text{Height}{\left(\mathrm{cm}\right)}^{0.310}$$

$${\mathrm{HI}}_{\mathrm{men}}=\mathrm{HC}\left(\mathrm{cm}\right)*\text{Weight}{\left(\mathrm{kg}\right)}^{-\frac{2}{5}}*\text{Height}{\left(\mathrm{cm}\right)}^{\frac{1}{5}}$$

For comparability, we transformed all anthropometric indices to sex‐specific *z*‐scores [value minus mean divided by standard deviation (SD)] and calculated pairwise Pearson correlation coefficients (*r*).

### 2.4 Statistical Analysis

We used STATA‐13 for the statistical analyses and R version 4.1.3 [23] for data management. Tests of statistical significance were two‐sided and were evaluated at a nominal cut‐off of *p* < 0.05.

We examined as exposure variables anthropometric indices and diabetes (any self‐reported or use of antidiabetic drugs, or HbA1c ≥ 48 mmol/mol, no/yes) in multivariable delayed‐entry Cox proportional hazards models, which are conditional on surviving free of cancer to cohort recruitment and thus account for left truncation. The underlying time scale was age (origin—date of birth; entry time—date at cohort recruitment; and exit time—the earliest of the following: date of diagnosis of the first primary incident cancer, date of death or date of end of cancer follow‐up). We interpreted hazard ratios (HR) (95% confidence intervals, 95% CI) as change per 1 SD increase for the anthropometric indices and as difference between individuals with diabetes compared to those without.

Covariates were selected a priori to include the major lifestyle, metabolic and dietary factors that are known to be associated with obesity and have been reported in association with pancreatic cancer risk. Pairwise associations of covariates with body composition, abdominal obesity and pancreatic cancer risk are shown in Figure S1 (references related to the selection are included in the legend). The fully adjusted models were stratified by age categories (40 to < 55 years jointly, due to the smaller number of pancreatic cancer cases in younger individuals, and thereafter in 5‐year groups: 55 to < 60, 60 to < 65 and 65–70 years at recruitment) and a combined variable of sex, menopausal status and hormone replacement therapy (HRT) use (men, premenopausal women, post/unknown menopause never used HRT, post/unknown menopause ever used HRT). The fully adjusted models also included as covariates the major lifestyle factors—smoking status (never, former occasional, former regular or current), alcohol consumption (≤ 3 times/month, ≤ 4 times/week or daily) and physical activity (less active, moderately active or very active). As proxies of socioeconomic status, we used education (primary school, secondary/vocational, university degree) and Townsend deprivation index (cohort‐specific tertiles). Covariates further included family history of cancer (no/yes for bowel, lung, prostate or breast cancer in parents or siblings, as no information was available for family history of pancreatic cancer); and factors related to the metabolic syndrome (because abdominal obesity and diabetes are features of the metabolic syndrome)—hypertension (any of self‐reported or use of antihypertensive drugs, no/yes), use of lipid‐lowering drugs (no/yes), nonsteroidal anti‐inflammatory drugs (no/yes; because these can influence inflammation and obesity is accompanied with low‐grade chronic inflammation) and antiaggregant/anticoagulants (no/yes; because these are prescribed to individuals with cardiovascular diseases and platelets are involved in immune inflammation). Last, covariates included dietary intake (low/high, dichotomised at the upper cohort‐specific tertile boundary) of fruit (> 3 portions/day); vegetables (> 5 portions/day); fibre (> 16 g/day); red meat (> 2 times/week); processed meat (> once/week); fish (> 2 times/week); tea (> 4 cups/day); and coffee (> 2 cups/day). Missingness in covariates was very low (< 2%), so we replaced missing values with the sex‐specific median category, as previously described [8]. For the definition of covariates and percentage missingness per covariate, see the legend of Figure S1.

To justify the use in the main analyses of the allometric body composition indices, which account for the relatedness between body composition measures and height prior to examining their associations with cancer risk, we compared in fully adjusted models the HR estimates for body composition and height when examined individually and after mutual adjustment, in joint models combining the corresponding alternative FM and FFM pairs (total, index, residual or allometric) with height. To justify the use in the main analyses of the allometric WHI, which accounts for the relatedness of waist and hip circumferences with BMI and height prior to examining associations with cancer risk, we compared HR estimates for alternative body shape indices with HR estimates for BMI.

We then examined jointly as exposure variables in models fully adjusted for covariates the complete set of anthropometric indices—AFI, ALI, WHI and height. For comparison, we also examined fully adjusted models including jointly as exposure variables the set of BMI, WHI and height, or the set of residual FM, residual FFM, WHI and height. We examined associations in participants overall, separately in women and men, in groups according to age (cut‐off at ≥ 55 years, which halves the UK Biobank range), and according to follow‐up time (cut‐off at ≥ 6 years, which is close to the mean follow‐up time of 6.5 years in pancreatic cancer cases). We tested for heterogeneity by sex, age and follow‐up time (p_sex_, p_age_ and p_follow‐up_) with the data augmentation method of Lunn and McNeil [24]. We additionally performed analyses in groups according to age and follow‐up time separately in women and men, corresponding to the sex‐specific analyses in our previous cancer‐wide study [8].

We performed the following sensitivity analyses. To examine the influence of covariates, we compared HR estimates from the fully adjusted models with HR estimates from unadjusted models (omitting all covariates and stratifying only by sex). As smoking and diabetes showed the most prominent pairwise associations with pancreatic cancer risk among covariates examined individually (Table S2), we additionally examined models adjusted only for smoking status and models adjusted for smoking status and diabetes (in either case stratified by sex).

## 3 Results

### 3.1 Cohort Characteristics

During a mean follow‐up time of 10.4 years (SD = 2.2), 999 pancreatic cancer cases (461 women; 538 men) were ascertained in 427,939 participants (230,404 women; 197,535 men). Women had lower BMI, total FFM and height, but higher total FM compared to men (Table 1). All waist indices were lower in women compared to men, while hip circumference was similar. Women were less likely to be current smokers and to have diabetes. Cases with shorter and longer follow‐up times did not differ materially.

**TABLE 1:** Characteristics of study participants.

Group	Participants overall	Participants women	Participants men	Cases overall	Cases FU < 6 years	Cases FU ≥ 6 years
Total number (%)	427,939	230,404 (53.8)	197,535 (46.2)	999	422 (42.2)	577 (57.8)
Follow‐up years^a^	10.4 (2.2)	10.5 (2.1)	10.3 (2.4)	6.5 (3.1)	3.4 (1.7)	8.7 (1.6)
Age years^a^	57.0 (8.0)	56.8 (8.0)	57.2 (8.1)	61.7 (6.3)	62.1 (6.0)	61.5 (6.4)
Body size^a^						
Height m	168.7 (9.3)	162.6 (6.2)	175.9 (6.8)	169.3 (9.3)	169.6 (9.4)	169.0 (9.2)
Weight kg	78.2 (15.9)	71.4 (13.9)	86.2 (14.2)	81.6 (16.9)	81.4 (16.8)	81.7 (17.0)
BMI kg/m^2^	27.4 (4.7)	27.0 (5.1)	27.9 (4.2)	28.4 (5.0)	28.2 (5.1)	28.5 (4.9)
Total FM kg	24.8 (9.5)	26.8 (10.0)	22.4 (8.2)	26.3 (10.2)	26.0 (10.5)	26.5 (9.9)
Total FFM kg	53.5 (11.6)	44.6 (5.0)	63.9 (7.7)	55.3 (12.0)	55.4 (11.8)	55.2 (12.1)
FM index	8.8 (3.6)	10.2 (3.8)	7.2 (2.7)	9.3 (3.8)	9.2 (4.0)	9.4 (3.7)
FFM index	18.6 (2.6)	16.9 (1.7)	20.6 (1.9)	19.1 (2.7)	19.1 (2.6)	19.1 (2.7)
Residual FM^£^	0 (9.2)	0 (9.9)	0 (8.2)	2.0 (9.8)	1.7 (9.9)	2.2 (9.7)
Residual FFM^£^	0 (3.8)	0 (3.0)	0 (4.6)	−0.2 (3.7)	−0.3 (3.6)	−0.2 (3.8)
AFI^£^	13.2 (4.9)	13.9 (5.1)	12.5 (4.6)	14.2 (5.3)	14.0 (5.5)	14.3 (5.2)
ALI^£^	14.5 (1.1)	14.5 (1.0)	14.6 (1.1)	14.5 (1.0)	14.5 (1.0)	14.5 (1.0)
Body shape^a^						
WC cm	90.2 (13.4)	84.4 (12.4)	97.0 (11.2)	94.1 (14.1)	93.9 (13.8)	94.3 (14.3)
AVI	16.8 (4.9)	14.9 (4.4)	19.1 (4.5)	18.3 (5.4)	18.1 (5.2)	18.3 (5.4)
WHtR	0.53 (0.07)	0.52 (0.08)	0.55 (0.06)	0.56 (0.08)	0.55 (0.08)	0.56 (0.08)
BRI	4.14 (1.57)	3.86 (1.65)	4.47 (1.41)	4.59 (1.68)	4.54 (1.70)	4.62 (1.67)
WHR	0.87 (0.09)	0.82 (0.07)	0.94 (0.06)	0.90 (0.09)	0.90 (0.09)	0.90 (0.10)
ConI	1.22 (0.10)	1.17 (0.09)	1.27 (0.07)	1.25 (0.1)	1.25 (0.1)	1.25 (0.1)
WWI	10.2 (0.8)	10.0 (0.8)	10.5 (0.6)	10.4 (0.8)	10.4 (0.8)	10.4 (0.8)
WHRadjBMI^£^	0 (0.06)	0 (0.06)	0 (0.05)	0.01 (0.06)	0.01 (0.06)	0.01 (0.06)
WHI	3.82 (0.35)	3.59 (0.27)	4.08 (0.22)	3.9 (0.36)	3.90 (0.35)	3.89 (0.37)
ABSI	76.6 (5.5)	73.8 (4.9)	79.8 (4.1)	77.9 (5.5)	78.0 (5.6)	77.8 (5.5)
HI^£^	57.3 (7.9)	64.3 (2.5)	49.1 (1.7)	56.1 (7.9)	55.9 (7.8)	56.3 (7.9)
HC cm	103.4 (9.1)	103.3 (10.3)	103.5 (7.5)	104.8 (9.9)	104.6 (10.1)	105.0 (9.7)
Smoking^b^						
Never	168,258 (39.3)	100,602 (43.7)	67,656 (34.3)	346 (34.6)	142 (33.6)	204 (35.4)
Former occasional	114,389 (26.7)	63,800 (27.7)	50,589 (25.6)	206 (20.6)	76 (18.0)	130 (22.5)
Former regular	100,712 (23.5)	45,551 (19.8)	55,161 (27.9)	295 (29.5)	129 (30.6)	166 (28.8)
Current	44,580 (10.4)	20,451 (8.9)	24,129 (12.2)	152 (15.2)	75 (17.8)	77 (13.3)
Alcohol consumption^b^						
≤ 3 times/month	121,358 (28.4)	81,167 (35.2)	40,191 (20.3)	281 (28.1)	122 (28.9)	159 (27.6)
≤ 4 times/week	216,314 (50.5)	110,701 (48.0)	105,613 (53.5)	461 (46.1)	180 (42.7)	281 (48.7)
Daily	90,267 (21.1)	38,536 (16.7)	51,731 (26.2)	257 (25.7)	120 (28.4)	137 (23.7)
Physical activity^b^						
Less active	68,854 (16.1)	38,695 (16.8)	30,159 (15.3)	191 (19.1)	74 (17.5)	117 (20.3)
Moderately active	209,923 (49.1)	120,792 (52.4)	89,131 (45.1)	489 (48.9)	214 (50.7)	275 (47.7)
Very active	149,162 (34.9)	70,917 (30.8)	78,245 (39.6)	319 (31.9)	134 (31.8)	185 (32.1)
Education^b^						
Primary school	71,428 (16.7)	38,228 (16.6)	33,200 (16.8)	247 (24.7)	107 (25.4)	140 (24.3)
Secondary/vocational	219,478 (51.3)	120,933 (52.5)	98,545 (49.9)	475 (47.5)	199 (47.2)	276 (47.8)
University degree	137,033 (32.0)	71,243 (30.9)	65,790 (33.3)	277 (27.7)	116 (27.5)	161 (27.9)
Townsend index^c^	−2.27 (3.94)	−2.27 (3.87)	−2.26 (4.01)	−2.17 (4.29)	−2.15 (4.37)	−2.17 (4.23)
Family history of cancer^d^	151,565 (35.4)	82,331 (35.7)	69,234 (35.0)	408 (40.8)	197 (46.7)	211 (36.6)
Diabetes^d^	22,317 (5.2)	8107 (3.5)	14,210 (7.2)	127 (12.7)	57 (13.5)	70 (12.1)
Hypertension^d^	121,011 (28.3)	56,331 (24.4)	64,680 (32.7)	405 (40.5)	170 (40.3)	235 (40.7)
Lipid‐lowering drugs^d^	74,337 (17.4)	28,749 (12.5)	45,588 (23.1)	311 (31.1)	127 (30.1)	184 (31.9)
NSAID^d^	79,681 (18.6)	49,199 (21.4)	30,482 (15.4)	171 (17.1)	69 (16.4)	102 (17.7)
Antiaggregants^d^	65,247 (15.2)	24,509 (10.6)	40,738 (20.6)	232 (23.2)	82 (19.4)	150 (26.0)
Dietary factors^d^						
Fruit > 3 portions/day	138,684 (32.4)	86,447 (37.5)	52,237 (26.4)	328 (32.8)	128 (30.3)	200 (34.7)
Veg > 5 portions/day	130,735 (30.5)	77,311 (33.6)	53,424 (27.0)	296 (29.6)	120 (28.4)	176 (30.5)
Fibre > 16 g/day	144,803 (33.8)	81,187 (35.2)	63,616 (32.2)	335 (33.5)	131 (31.0)	204 (35.4)
Red meat > 2 times/week	135,310 (31.6)	65,115 (28.3)	70,195 (35.5)	363 (36.3)	149 (35.3)	214 (37.1)
Processed meat > once/week	135,425 (31.6)	48,220 (20.9)	87,205 (44.1)	345 (34.5)	150 (35.5)	195 (33.8)
Fish > 2 times/week	119,971 (28.0)	66,453 (28.8)	53,518 (27.1)	277 (27.7)	118 (28.0)	159 (27.6)
Tea > 4 cups/day	131,591 (30.7)	70,309 (30.5)	61,282 (31.0)	292 (29.2)	129 (30.6)	163 (28.2)
Coffee > 2 cups/day	140,589 (32.9)	69,478 (30.2)	71,111 (36.0)	347 (34.7)	147 (34.8)	200 (34.7)
Sex‐MP‐HRT^d^						
Men	197,535 (46.2)	—	197,535 (100)	538 (53.9)	232 (55.0)	306 (53.0)
Pre‐MP women	55,505 (13.0)	55,505 (24.1)	—	28 (2.8)	12 (2.8)	16 (2.8)
Post‐MP^#^ never HRT	87,104 (20.4)	87,104 (37.8)	—	173 (17.3)	74 (17.5)	99 (17.2)
Post‐MP^#^ ever HRT	87,795 (20.5)	87,795 (38.1)	—	260 (26.0)	104 (24.6)	156 (27.0)

*Note:* Comparisons between sexes and age groups were performed with unpaired‐samples t‐test (for continuous variables), Wilcoxon signed‐rank test (for Townsend index) and Chi‐squared test (for categorical variables). All comparisons between women and men were statistically significant at *p* < 0.0001, except for Townsend index (*p* = 0.003) and tea consumption (*p* = 0.0003). No comparisons between cases with shorter and longer follow‐up times were statistically significant, except for family history of cancer (*p* = 0.002) and use of antiaggregants/anticoagulants (*p* = 0.019).

Abbreviations: ABSI, a body shape index; AFI, allometric fat‐mass index; ALI, allometric lean‐mass index; AVI, abdominal volume index; BMI, body mass index; BRI, body roundness index; ConI, conicity index; FFM, fat‐free mass; FM, fat mass; FM & FFM index, total FM or total FFM (kg) divided by height (m) squared; FU, follow‐up time; HC, hip circumference; HI, hip index; MP, menopause (^#^Post‐MP includes also women with undetermined/unknown menopausal status); Residual FM & FFM, residuals of total FM (kg) regressed on height (cm) or total FFM (kg) regressed on height (cm) and total FM (kg). Total FM & FFM, measured with bioelectrical impedance; WC, waist circumference; WHI, waist‐to‐hip index; WHR, waist‐to‐hip ratio; WHRadjBMI, waist‐to‐hip ratio adjusted for BMI (residuals of WHR regressed on BMI); WHtR, waist‐to‐height ratio.

^a^ Mean (standard deviation).

^b^ Number per category (% from total per column).

^c^ Median (interquartile range).

^d^ Number for yes (% from total per column).

^£^ Calculated separately in women and men.

### 3.2 Correlations Between Anthropometric Indices

**FIGURE 1:**
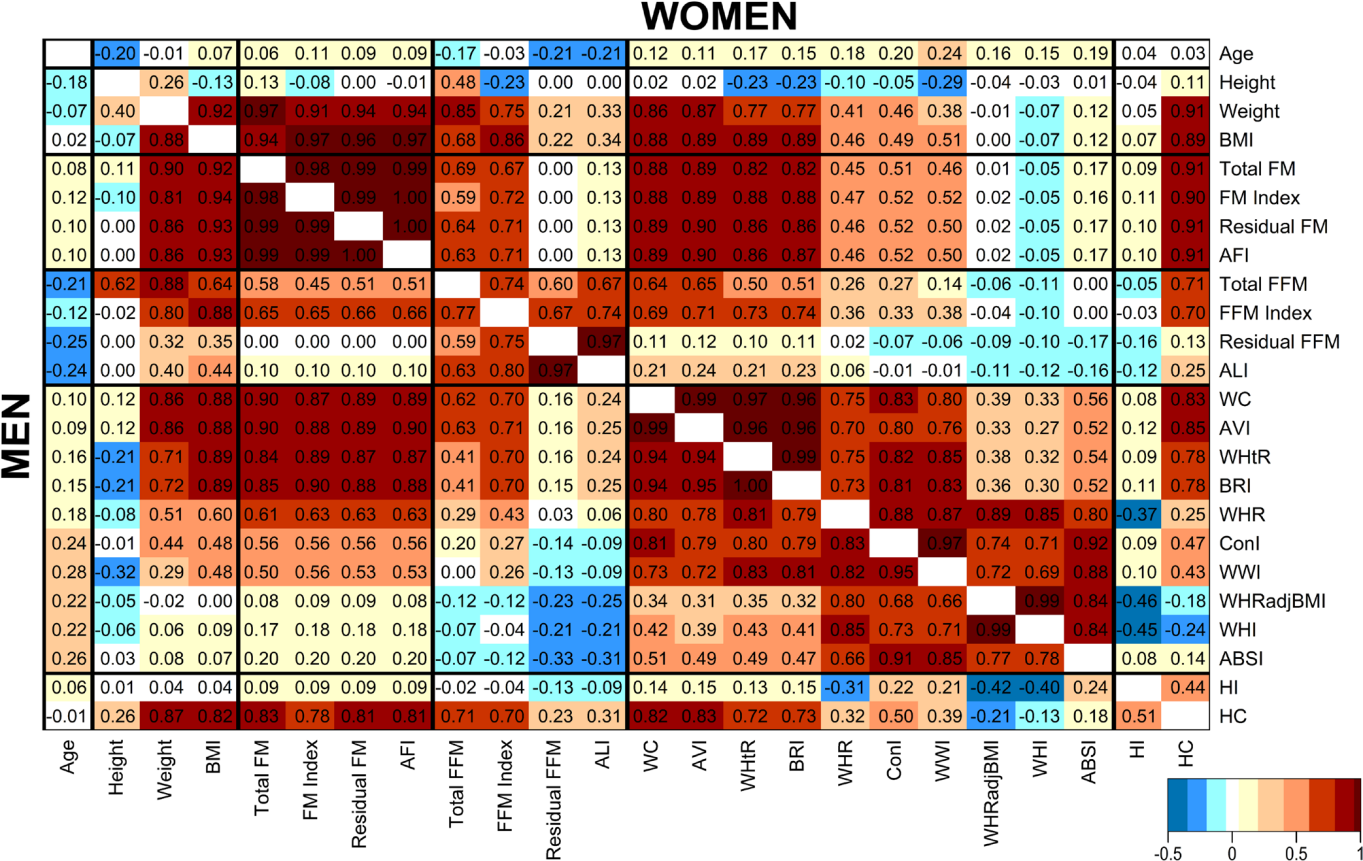
Pairwise correlations of body size and body shape indices. ABSI, a body shape index; AFI, allometric fat‐mass index; ALI, allometric lean‐mass index; AVI, abdominal volume index; BMI, body mass index; BRI, body roundness index; ConI, conicity index; FM, fat mass; FFM, fat‐free mass; HC, hip circumference; HI, hip index; WC, waist circumference; WHI, waist‐to‐hip index; WHR, waist‐to‐hip ratio; WHRadjBMI, waist‐to‐hip ratio adjusted for BMI (residuals of WHR regressed on BMI); WHtR, waist‐to‐height ratio; WWI, weight‐adjusted waist index. Cell values represent Pearson correlation coefficients. Total FM and total FFM were measured with bioelectrical impedance. FM Index and FFM Index were calculated by dividing total FM and total FFM (kg), correspondingly, by height (m) squared. Residual FM and residual FFM were derived in this study (separately for women and men) as the corresponding residuals of total FM (kg) regressed on height (cm) or total FFM (kg) regressed on height (cm) and total FM (kg). All anthropometric indices were transformed to sex‐specific *z*‐scores (value minus mean divided by standard deviation).

In women and men, height and FFM alternatives (total, index, residual and ALI) were inversely correlated with age, most strongly for ALI and residual FFM, while all FM alternatives were weakly positively correlated with age (Figure 1). Weight was positively correlated with height, but BMI was weakly inversely correlated with height. FM alternatives were correlated weakly with height but very strongly positively with BMI (*r* > 0.90). Residual FM and AFI were effectively identical (*r* = 1.00). Total FFM was correlated substantially positively with height and with FM alternatives. FFM index was uncorrelated with height in men but was inversely correlated in women and in both sexes was substantially positively correlated with FM alternatives. Residual FFM and ALI were effectively identical (*r* > 0.97) and were uncorrelated with height. Residual FFM was uncorrelated with FM alternatives, and ALI was only weakly positively correlated with them (*r* ≈ 0.10).

All waist indices were positively correlated with age (Figure 1). Waist circumference and AVI were effectively identical (*r* > 0.99) and WHtR and BRI were effectively identical (*r* > 0.99), and they were all very strongly positively correlated with BMI and weight, but only WHtR and BRI were inversely correlated with height. WHR, ConI and WWI were moderately positively correlated with BMI and weight, but only WWI was correlated more substantially inversely with height. WHRadjBMI and WHI were effectively identical (*r* > 0.99) and both, as well as ABSI and HI, were correlated little with BMI, weight and height. While HI was correlated inversely with WHR, WHRadjBMI and WHI, hip circumference was not only strongly positively correlated with BMI, like waist circumference, but was also positively correlated with WHR.

### 3.3 Associations of Alternative Body Composition Indices With Pancreatic Cancer Risk

**FIGURE 2:**
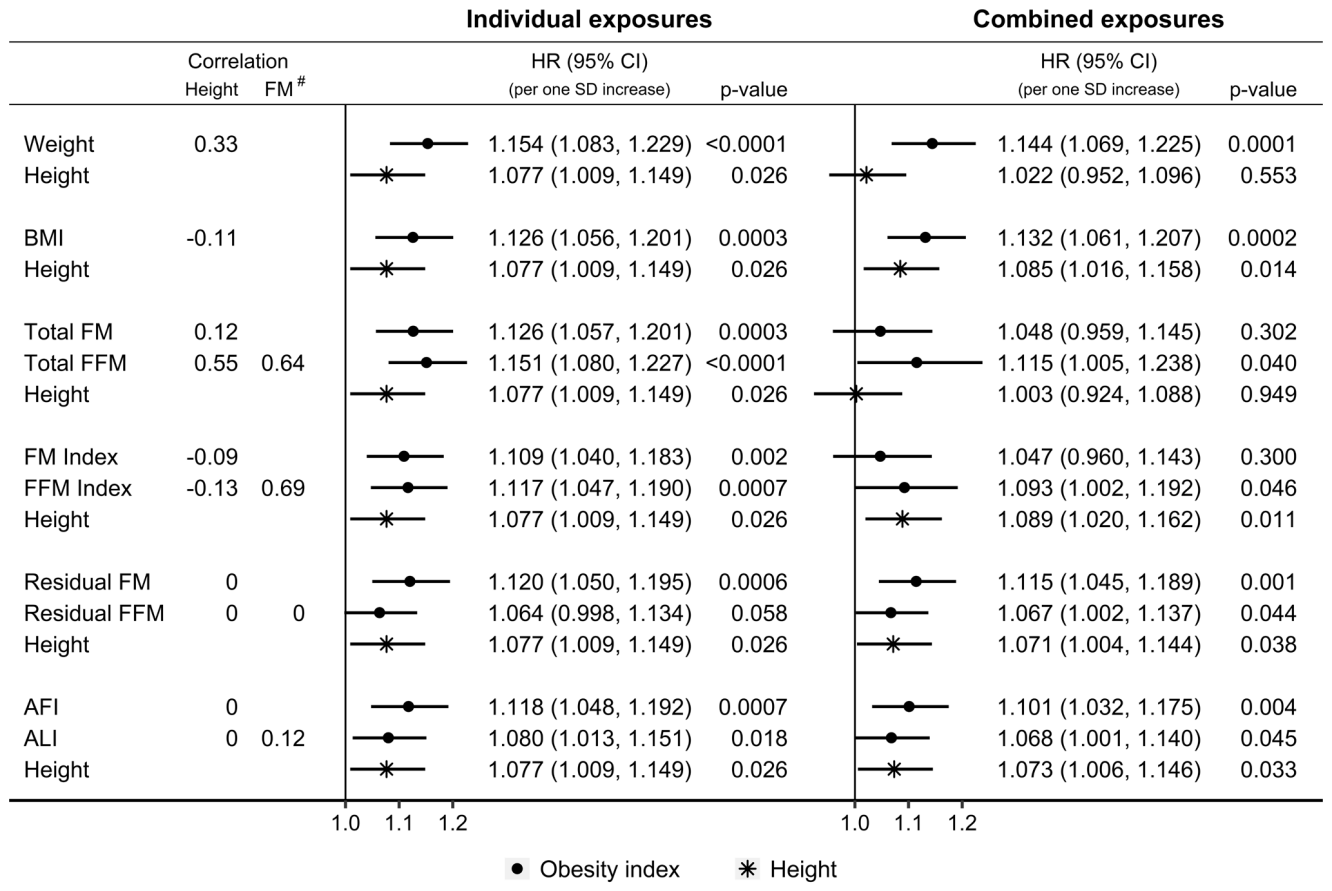
Associations of alternative body composition indices with pancreatic cancer risk. AFI, allometric fat‐mass index; ALI, allometric lean‐mass index; BMI, body mass index; CI, confidence interval; FM, fat mass; FFM, fat‐free mass; HR, hazard ratio; SD, standard deviation; Total FM & FFM, measured with bioelectrical impedance (kg); FM & FFM index, total FM or FFM (kg) divided by height (m) squared; Residual FM & FFM, residuals of total FM (kg) regressed on height (cm) or total FFM (kg) regressed on total FM (kg) and height (cm); Correlation, pairwise partial Pearson correlation coefficient adjusted for sex (^#^ pairwise correlation between the corresponding FM and FFM pair); *p* value, Wald test for the individual term. Individual exposures—estimates from Cox proportional hazards models in participants overall, including individually as exposure variable one of the listed anthropometric measures or indices (sex‐specific *z*‐scores, value minus mean divided by SD), stratified by age and a combined variable of sex, menopausal status and hormone replacement therapy use, and adjusted for smoking status, alcohol consumption, physical activity, education, Townsend deprivation index, family history of cancer, diabetes, hypertension, use of lipid‐lowering drugs, nonsteroidal anti‐inflammatory drugs and antiaggregant/anticoagulants, and dietary intake of fruit, vegetables, fibre, red meat, processed meat, fish, tea and coffee. Combined exposures—Fully adjusted models (as described above), including jointly the obesity measures and indices listed in each group and height.

Both weight and height were positively associated with pancreatic cancer risk when examined individually in fully adjusted models, but when combined in the same model, the association of height with pancreatic cancer risk was lost (Figure 2). On the contrary, both BMI and height were positively associated with pancreatic cancer risk when combined, as well as when examined individually.

Total FM and total FFM showed similar positive associations with pancreatic cancer risk when examined individually, but combining them with height attenuated their associations (more prominently for total FM) and widened their confidence intervals (more prominently for total FFM), and additionally abolished the positive association of height with pancreatic cancer risk (Figure 2). FM index and FFM index also showed similar positive associations with pancreatic cancer risk when examined individually, but combining them with height attenuated only their associations, without materially affecting the positive association of height with pancreatic cancer risk. On the contrary, residual FM and residual FFM, as well as height, were positively associated with pancreatic cancer risk when combined, as well as when examined individually. Similarly, AFI, ALI and height remained positively associated with pancreatic cancer risk when combined, as well as when examined individually.

### 3.4 Associations of Alternative Body Shape Indices With Pancreatic Cancer Risk

**FIGURE 3:**
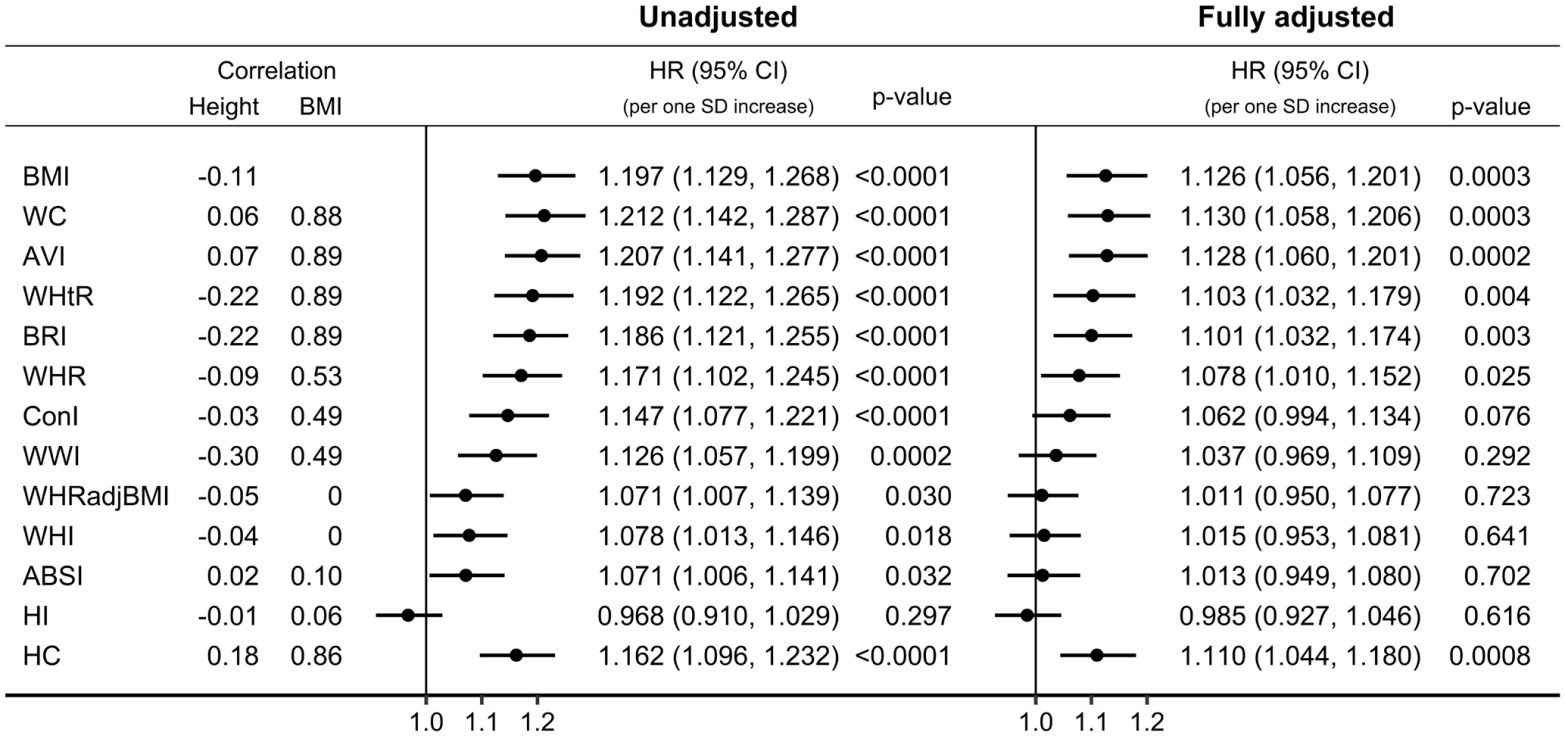
Associations of alternative body shape indices with pancreatic cancer risk. ABSI, a body shape index; AVI, abdominal volume index; BMI, body mass index; BRI, body roundness index; CI, confidence interval; ConI, conicity index; HC, hip circumference; HI, hip index; HR, hazard ratio; SD, standard deviation; WC, waist circumference; WHI, waist‐to‐hip index; WHR, waist‐to‐hip ratio; WHRadjBMI, waist‐to‐hip ratio adjusted for BMI (residuals of WHR regressed on BMI); WHtR, waist‐to‐height ratio; WWI, weight‐adjusted waist index; Correlation, pairwise partial Pearson correlation coefficient adjusted for sex; *p* value, Wald test for the individual term. Unadjusted—Estimates from unadjusted Cox proportional hazards models in participants overall (stratified only by sex), including individually as exposure variable one of the listed body shape indices (sex‐specific *z*‐scores, value minus mean divided by SD). BMI is shown for comparison. Fully adjusted—As above but stratified by age and a combined variable of sex, menopausal status and hormone replacement therapy use, and adjusted for smoking status, alcohol consumption, physical activity, education, Townsend deprivation index, family history of cancer, diabetes, hypertension, use of lipid‐lowering drugs, nonsteroidal anti‐inflammatory drugs and antiaggregant/anticoagulants, and dietary intake of fruit, vegetables, fibre, red meat, processed meat, fish, tea and coffee.

In unadjusted models, stratified only by sex, all waist indices were positively associated with pancreatic cancer risk, with effect sizes proportional to their correlations with BMI—strongest for waist circumference and AVI and weakest for WHRadjBMI, WHI and ABSI (Figure 3). Although waist circumference, AVI, WHtR and BRI were similarly positively correlated with BMI, the positive associations of WHtR and BRI with pancreatic cancer risk were slightly weaker, corresponding to their inverse correlations with height. Although ConI and WWI were similarly correlated with BMI, the positive association of WWI with pancreatic cancer risk was slightly weaker, corresponding to its inverse correlation with height. In fully adjusted models, the associations of all waist indices with pancreatic cancer risk were attenuated and were lost for WHRadjBMI, WHI and ABSI. HI was not associated with pancreatic cancer risk, irrespective of the adjustment for covariates, but hip circumference, like waist circumference, was positively associated with pancreatic cancer risk, more strongly in the unadjusted model.

The attenuation of the positive association of waist indices with pancreatic cancer risk was mostly accounted for by the adjustment for diabetes (for waist indices strongly correlated with BMI) or by the adjustment for smoking status and diabetes (for waist indices showing moderate or no correlations with BMI) (Figure S2).

### 3.5 Independent Associations of Anthropometric Indices and Diabetes With Pancreatic Cancer Risk

**FIGURE 4:**
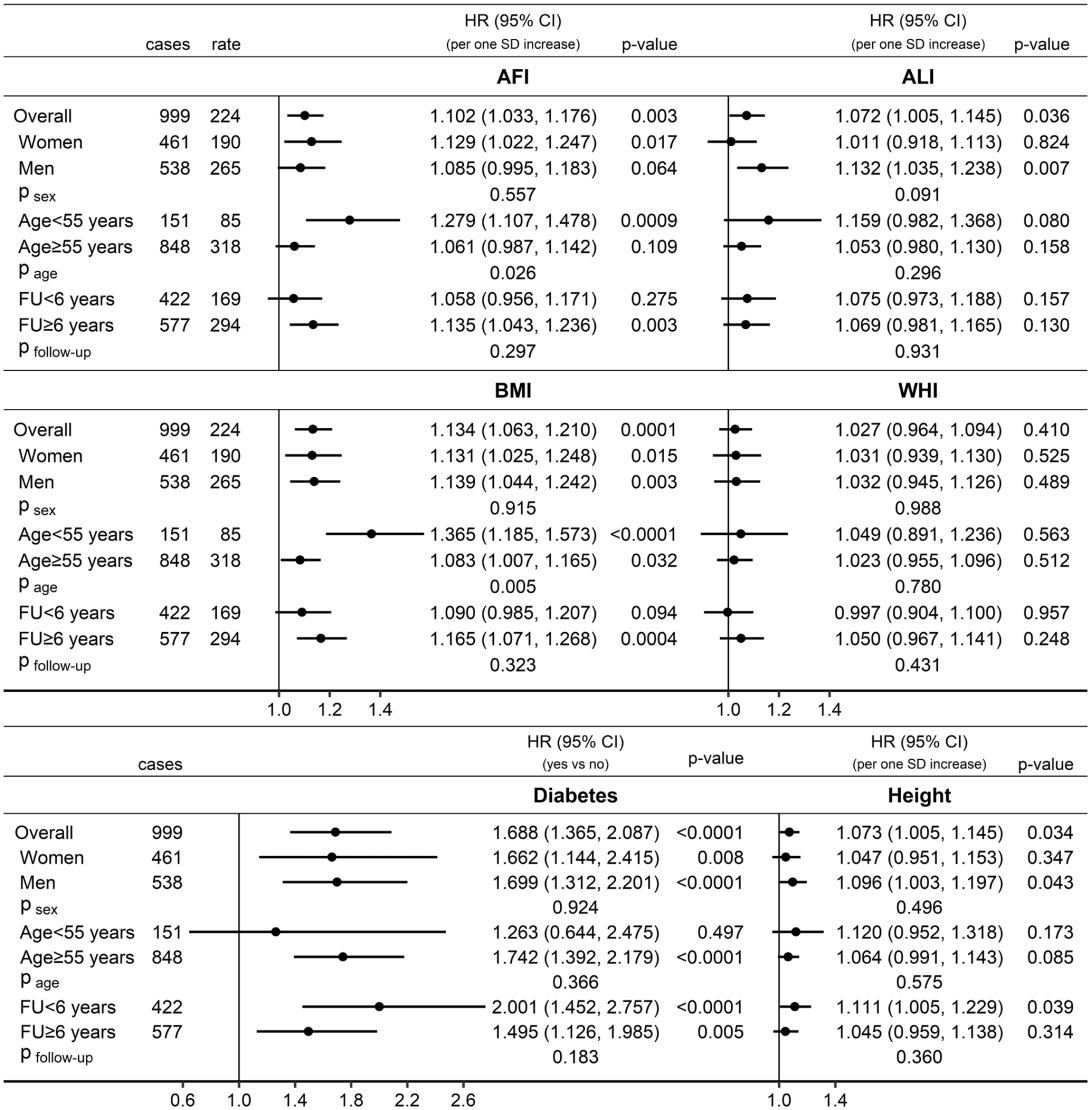
Independent associations of anthropometric indices and diabetes with pancreatic cancer risk. AFI, allometric fat‐mass index; ALI, allometric lean‐mass index; BMI, body mass index; CI, confidence interval; FU, follow‐up time; HR, hazard ratio; SD, standard deviation; WHI, waist‐to‐hip index (allometric); Cases, number of pancreatic cancer cases per group; Rate, incidence rate per 1 × 10^6^ person‐years in each group; *p* value, Wald test for the individual term. Estimates from multivariable Cox proportional hazards models including jointly as exposure variables AFI, ALI, WHI, height (sex‐specific *z*‐scores, value minus mean divided by SD) and diabetes, stratified by age and a combined variable of sex, menopausal status and hormone replacement therapy use, and adjusted for smoking status, alcohol consumption, physical activity, education, Townsend deprivation index, family history of cancer, hypertension, use of lipid‐lowering drugs, nonsteroidal anti‐inflammatory drugs and antiaggregant/anticoagulants, and dietary intake of fruit, vegetables, fibre, red meat, processed meat, fish, tea and coffee. Estimates for BMI were obtained from similar joint fully adjusted models including BMI instead of AFI and ALI. *p*_sex_/*p*_age_/*p*_follow‐up_—*p* value obtained with the data augmentation method of Lunn and McNeil [24] for the comparison of HR estimates between the specified groups according to sex (women, men), age at recruitment (< 55 years, ≥ 55 years) and follow‐up time (< 6 years, ≥ 6 years).

In fully adjusted models (examining jointly AFI, ALI, WHI, height and diabetes), AFI was positively associated with pancreatic cancer risk in participants overall (HR = 1.102; 95% CI = 1.033–1.176 per 1 SD increase), similarly in women and men (*p*_sex_ = 0.557), and with little difference according to follow‐up time (*p*_*f*ollow‐up_ = 0.297), but more strongly in participants aged < 55 years at recruitment (HR = 1.279; 95% CI = 1.107–1.478) than in older participants (HR = 1.061; 95% CI = 0.987–1.142, p_age_ = 0.026) (Figure 4). BMI showed a similar association pattern to AFI. ALI was also positively associated with pancreatic cancer risk in participants overall (HR = 1.072; 95% CI = 1.005–1.145 per 1 SD increase), more specifically in men (HR = 1.132; 95% CI = 1.035–1.238) but not in women (HR = 1.011; 95% CI = 0.918–1.113, p_sex_ = 0.091), with little evidence for heterogeneity by age (p_age_ = 0.296) or follow‐up time (p_follow‐up_ = 0.931). There was no evidence for independent associations of WHI with pancreatic cancer risk. Diabetes was positively associated with pancreatic cancer risk in participants overall HR = 1.688; 95% CI = 1.365–2.087 for diabetes yes vs. no), with little evidence for heterogeneity by sex (p_sex_ = 0.924), age (p_age_ = 0.366) or follow‐up time (p_follow‐up_ = 0.183). Height was also positively associated with pancreatic cancer risk in participants overall (HR = 1.073; 95% CI = 1.005–1.145 per 1 SD increase), with no evidence for heterogeneity by sex (p_sex_ = 0.496), age (p_age_ = 0.575) or follow‐up time (p_follow‐up_ = 0.360).

**FIGURE 5:**
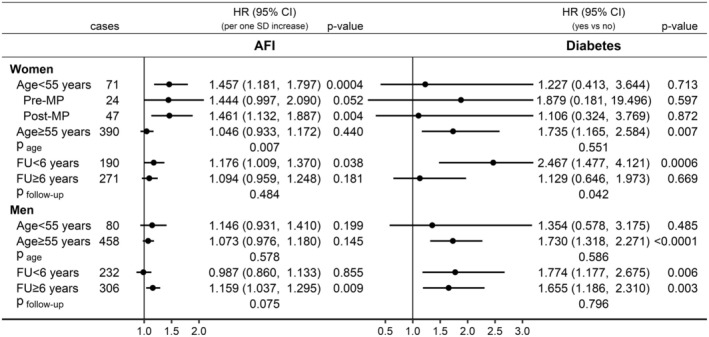
Independent associations of allometric fat‐mass index and diabetes with pancreatic cancer risk in women and men. AFI, allometric fat‐mass index; CI, confidence interval; FU, follow‐up time; HR, hazard ratio; Pre‐MP, women premenopausal at recruitment; Post‐MP, women postmenopausal at recruitment or with unknown/undetermined menopausal status; SD, standard deviation; Cases, number of pancreatic cancer cases per group; *p* value—Wald test for the individual term. Estimates from multivariable Cox proportional hazards models including jointly as exposure variables AFI, allometric lean‐mass index, waist‐to‐hip index, height (sex‐specific *z*‐scores, value minus mean divided by SD) and diabetes, stratified by age and a combined variable of sex, menopausal status and hormone replacement therapy use, and adjusted for smoking status, alcohol consumption, physical activity, education, Townsend deprivation index, family history of cancer, hypertension, use of lipid‐lowering drugs, nonsteroidal anti‐inflammatory drugs and antiaggregant/anticoagulants, and dietary intake of fruit, vegetables, fibre, red meat, processed meat, fish, tea and coffee. The upper bound for diabetes was truncated at 3.2. Sex‐specific associations of body mass index, allometric lean‐mass index, waist‐to‐hip index and height with pancreatic cancer risk are shown in Figure S3. *p*_age_/*p*_follow‐up_—*p* value obtained with the data augmentation method of Lunn and McNeil [24] for the comparison of HR estimates between the specified groups according to age at recruitment (< 55 years, ≥ 55 years) and follow‐up time (< 6 years, ≥ 6 years).

Specifically in women, AFI was positively associated with pancreatic cancer risk only for age < 55 years at recruitment (HR = 1.457; 95% CI = 1.181–1.797), with no material difference according to menopausal status, but not for older age (HR = 1.046; 95% CI = 0.933–1.172; p_age_ = 0.007) (Figure 5). Also specifically in women, diabetes was more strongly positively associated with pancreatic cancer risk for follow‐up < 6 years (HR = 2.467; 95% CI = 1.477–4.121) and not for longer follow‐up time (HR = 1.129; 95% CI = 0.646–1.973, p_follow‐up_ = 0.042). Specifically in men, AFI was positively associated with pancreatic cancer risk for follow‐up ≥ 6 years (HR = 1.159; 95% CI = 1.037–1.295) but not for shorter follow‐up time (HR = 0.987; 95% CI = 0.860–1.133; p_follow‐up_ = 0.075). BMI showed similar association patterns with pancreatic cancer risk to AFI but there was no evidence for heterogeneity by age or follow‐up time for the associations of ALI, WHI and height with pancreatic cancer risk in the sex‐specific analyses (Figure S3).

The association patterns of residual FM and residual FFM with pancreatic cancer risk, examined jointly with WHI, height and diabetes in fully adjusted models, resembled the corresponding association patterns of AFI and ALI (Figure S4).

### 3.6 Sensitivity Analyses

In unadjusted models (examining jointly AFI, ALI, WHI and height, with stratification by sex), the positive associations of AFI with pancreatic cancer risk were stronger, with the adjustment for diabetes accounting for most of the difference from the fully adjusted models, but with no material influence of the adjustment for smoking and little influence of further adjustment for covariates (Figure S5). The positive associations of ALI with pancreatic cancer risk were influenced little by adjustment for covariates. The association patterns of residual FM and residual FFM resembled the corresponding association patterns of AFI and ALI. WHI was positively associated with pancreatic cancer risk in the unadjusted joint models, similarly to when examined individually, and the adjustment for smoking and diabetes accounted for most of the complete attenuation in the fully adjusted models. The positive associations of diabetes with pancreatic cancer risk were stronger in unadjusted models (examining diabetes individually, with stratification by sex) and adjustment for anthropometric indices accounted for most of the differences from the fully adjusted models (largest attenuation in participants aged < 55 years at recruitment), while the positive associations of height with pancreatic cancer risk were influenced little by adjustment for covariates.

## 4 Discussion

Our findings of a positive association of general obesity (AFI and BMI) with pancreatic cancer risk are consistent with prior large‐scale prospective studies examining associations of BMI [7, 25] or polygenic risk scores for BMI with pancreatic cancer risk, including an attenuation after adjustment for diabetes [9]. We have additionally shown, however, that the associations of AFI and BMI with pancreatic cancer risk were stronger in younger individuals, when pancreatic cancer is less common, and obesity would more likely be general than abdominal and would more likely be genetically determined [26]. Correspondingly, high BMI in early adulthood has been associated with higher pancreatic cancer risk in later life [27, 28, 29], although with some indication for a stronger association in men, while we found a pronounced age difference in women. One explanation for the latter discrepancy could be that we have examined age and sex differences at cohort recruitment (not in early life), which would be closer to diagnosis. In addition to the observational studies, several MR studies have reported positive associations of genetically proxied BMI and FM index with pancreatic cancer risk [30, 31, 32], with a direct effect of BMI, as well as a mediation via fasting insulin [32], thus supporting a causal effect of general obesity on pancreatic cancer risk independent of diabetes.

Further supporting the role of general fat accumulation in pancreatic cancer development, the risk of pancreatic cancer in humans is higher for higher prediagnostic total fat intake [33] and lower after weight loss following bariatric surgery [34]. In mice, a high‐fat diet contributes to the development of a more aggressive pancreatic cancer phenotype [35]. Fat accumulation specifically in the pancreas is more common among patients with pancreatic cancer [36] and in animal models facilitates pancreatic carcinogenesis [37]. In addition, independent of diabetes and BMI in humans, adiponectin, which is lower in obesity, is associated inversely with pancreatic cancer risk, while leptin, which is upregulated in obesity, is associated positively with pancreatic cancer risk; and, in animal models, adiponectin inhibits while leptin stimulates pancreatic cancer growth (reviewed in [38]). Furthermore, lipocalin 2, another adipokine upregulated in obesity, is a marker of pancreatic cancer [39] and, when depleted in a mouse model, reduces pancreatic cell growth [40]. Besides adipokines, a leptin‐independent mechanism suppressible by weight loss has been demonstrated in leptin‐deficient obese mice, in which an aberrant expression of the intestinal peptide hormone cholecystokinin in pancreatic beta cells enhances pancreatic cancer growth [41].

Independent of general and abdominal obesity, diabetes was also clearly positively associated with pancreatic cancer risk in our study, in agreement with large‐scale meta‐analyses of observational and MR studies, including in studies adjusting for BMI and smoking [42, 43, 44, 45]. Higher HbA1c and higher fasting or random glucose have also been associated with higher pancreatic cancer risk independent of BMI and in the nondiabetic range [43, 46, 47]. These associations are mechanistically plausible because high insulin in type 2 diabetes and insulin‐like growth factors, each via their own receptor and interchangeably, promote pancreatic cell growth [48]. Advanced glycation end‐products [49] and activation of the AKT/mTOR pathway in type 2 diabetes [50] can also promote pancreatic cancer development. Hyperglycaemia induces in pancreatic ductal epithelial cells epithelial–mesenchymal transition and acquisition of stem cell properties, which are essential for the initiation and maintenance of tumour growth [51, 52]. The latter process is further facilitated by inflammatory cytokines released from macrophages, which accompany the chronic low‐grade inflammation of obesity [52]. Furthermore, it has been proposed that pancreatic cancer and diabetes have a shared genetic background, with a common pathogenesis concerning the regulation of endodermal cell fate specification [53].

Besides the associations with general obesity and diabetes, we have shown that all waist indices were positively associated with pancreatic cancer risk, irrespective of whether they were simple ratios or were based on geometric or allometric considerations, but only in unadjusted models, with risk estimates proportional to the correlation of the waist index with BMI. After accounting for smoking and diabetes, only waist indices correlated with BMI remained positively associated with pancreatic cancer risk, in agreement with large meta‐analyses of prospective studies examining the associations of waist circumference and WHR with pancreatic cancer risk [7, 54]. Smoking is associated with both abdominal obesity [55] and higher pancreatic cancer risk [56] and thus may represent a confounder or may contribute to pancreatic cancer development by inducing abdominal obesity. Diabetes is also associated with abdominal obesity, as part of the metabolic syndrome [6], and with higher pancreatic cancer risk [42] and thus may be a confounder but may also be induced by the insulin resistance related to visceral fat accumulation. Nevertheless, no positive association remained for waist indices uncorrelated with BMI after accounting for smoking and diabetes, in agreement with previous studies in UK Biobank examining the observational associations of ABSI or polygenic risk scores for WHRadjBMI with pancreatic cancer risk [9, 57].

After factoring out associations with FM and height and thus removing the relatedness of FFM to obesity [11] and accounting for the fact that FFM measurements depend on body hydration and are overestimated in obesity [58], ALI was positively associated with pancreatic cancer risk, more specifically in men. Although there is no immediately obvious mechanistic explanation for this association, a positive association of red meat consumption with pancreatic cancer risk, independent of BMI and diabetes, has similarly been reported only for men and not for women [59]. The protein content of muscle mass could be relevant to pancreatic cancer development because pancreatic cancer cells, in both animal models and humans, source amino acids from extracellular proteins scavenged via macropinocytosis [60, 61]. Independent of ALI, height remained positively associated with pancreatic cancer risk, consistent with previous observational prospective studies [13].

Two major conclusions emerge from our findings related to indices of general and abdominal obesity. First, to define abdominal obesity as an entity different from general obesity and independent of height, an index of waist size should be used that is uncorrelated with BMI and uncorrelated with height. We have shown that this condition is fulfilled only by the allometric indices ABSI and WHI and by WHRadjBMI. All other alternative waist indices were associated with pancreatic cancer risk because they were positively associated with BMI, which reflects general obesity; hence, they do not provide information specific to abdominal obesity. The benefits for clinical practice of using ABSI or WHI are that they are free‐standing indices for which cut‐offs can be defined and individual patients can be classified with respect to them. The same argument applies to the allometric body composition indices AFI and ALI. The downside of ABSI, WHI, AFI and ALI is that they have been developed in populations with a white ethnic background and their power coefficients may not be translatable to populations with other ethnicities, thus permitting residual correlations and requiring recalibration of the power coefficients. While generating residuals as in WHRadjBMI, residual FM or residual FFM would completely factor out correlations in the examined dataset, this approach is more appropriate to population‐level studies and not to individual patients in clinical practice. The analogy with weight is unavoidable—the allometric index BMI has found widespread application in clinical practice and epidemiological studies, not the residuals of weight regressed on height or weight adjusted for height in the same model. The second major conclusion of our study concerns specifically pancreatic cancer. The association with general but not with abdominal obesity supports the hope that interventions aimed at regulating energy balance (reducing energy intake and increasing physical activity) can reduce pancreatic cancer risk. This is useful for public health because reducing the risk related to abdominal obesity is a harder task since less is known regarding how to induce a beneficial fat distribution. Individuals with abdominal obesity maintaining a healthy body weight will have a higher pancreatic cancer risk only if they develop diabetes. Individuals with diabetes, however, would be at a higher risk of pancreatic cancer even if they have a healthy body weight since diabetes and general obesity are independent risk factors.

Temporality, which provides insights into the influence of reverse causality, showed prominent sexual dimorphisms in our study. The positive association of AFI but not that of ALI with pancreatic cancer risk was lost closer to diagnosis in men, which agrees with the known high frequency of cachexia among pancreatic cancer patients, presenting with a greater loss of adipose than muscle tissue [62]. Pancreatic cancer cells induce cachexia via a feed‐forward loop involving interleukine‐6 signalling to which adipose tissue is more sensitive than skeletal muscle [63]. Furthermore, in animal models, early cachexia is more prominent in male compared to female mice [64], in agreement with our findings. Only in women, however, the positive association of diabetes with pancreatic cancer risk was stronger closer to diagnosis, potentially indicating that women with pancreatic cancer are more likely to develop cancer‐induced diabetes. Although the contribution of pancreatic cancer to the development of type 2 diabetes is well known [5, 65], no sex differences in the association of diabetes with pancreatic cancer risk have previously been found [44, 45] but sex differences in temporality have not been previously examined. Our study, however, supports a heightened pancreatic cancer alert in clinical practice for men with unintended weight loss and women with new‐onset type 2 diabetes.

Among the major strengths of our study are the prospective cohort design, the availability of body composition as well as standardised anthropometric measurements for the total cohort, and the sizable number of incident pancreatic cancer cases, which allowed us to examine heterogeneity by sex, age and follow‐up time. We have factored out associations between highly correlated and related anthropometric measurements prior to the statistical analysis, thus avoiding bias from their mutual adjustment in a joint statistical model. We have also compared a wide range of alternative waist indices illustrating their similarity and differences to BMI and general obesity. Limitations of our study are the lack of individuals younger than 40 and older than 70 years at recruitment, or information for weight in early adulthood. Most UK Biobank participants have white ancestry, preventing us from examining ethnic variations, and have healthier lifestyles compared to the general population, potentially limiting the range of the examined exposures. We could not use imaging body composition measurements because these were available only for 10% of participants and were assessed some 7 years after recruitment, thus limiting the number of incident pancreatic cancer cases. We did not have information about adipokine levels. Lastly, exposures and confounders were assessed only at recruitment, so we could not account for changes during follow‐up and for incident new‐onset diabetes.

## 5 Conclusions

General obesity (reflected in AFI and ALI) and diabetes were associated positively with pancreatic cancer risk, independent of each other and covariates. The positive associations with AFI were more prominent at a younger age in women, identifying them as the most important target group for public health interventions, and for a longer follow‐up time in men, identifying them as the group with more prominent cancer‐related cachexia. The positive association with diabetes was more prominent closer to diagnosis in women, identifying them as the group with more prominent cancer‐related diabetes induction. No independent association of abdominal obesity with pancreatic cancer risk remained after factoring out the associations of waist size with general obesity and height and accounting for smoking and diabetes.

## Author Contributions

**Sofia Christakoudi:** conceptualization (lead), data curation (lead), formal analysis (lead), investigation (equal), methodology (lead), project administration (lead), visualization (lead), writing – original draft (lead), writing – review and editing (equal). **Konstantinos K. Tsilidis:** conceptualization (equal), investigation (equal), methodology (equal), resources (supporting), supervision (equal), visualization (supporting), writing – original draft (supporting), writing – review and editing (equal). **Marc J. Gunter:** conceptualization (equal), investigation (equal), methodology (equal), resources (supporting), supervision (equal), visualization (supporting), writing – original draft (supporting), writing – review and editing (equal). **Elio Riboli:** conceptualization (equal), funding acquisition (lead), investigation (equal), methodology (equal), resources (lead), supervision (lead), writing – original draft (supporting), writing – review and editing (equal).

## Ethics Statement

This research was conducted according to the principles expressed in the Declaration of Helsinki. The UK Biobank cohort has been approved by the North West Multicenter Research Ethics Committee, UK (Ref: 16/NW/0274). Written informed consent has been obtained from all study participants. The current study was approved by the UK Biobank access management board. Participants who had withdrawn consent by the time of the analysis were excluded from the analysis dataset.

## Conflicts of Interest

The authors declare no conflicts of interest.

## Supporting information


Data S1.

## Data Availability

The dataset analysed in the current study was used under license and cannot be made freely available in a public repository or obtained from the authors due to restrictions related to privacy regulations and informed consent of the participants. Access to the data, however, can be obtained by bona fide researchers from UK Biobank, subject to approval of the research project and a material transfer agreement. For information on how to apply for gaining access to UK Biobank data, please follow the instructions at https://www.ukbiobank.ac.uk/enable‐your‐research. Further queries related to the data could be addressed to the corresponding author Dr Sofia Christakoudi s.christakoudi@imperial.ac.uk.
