# Supplementary material for: Prospective Associations of Body Composition and Body Shape With the Risk of Developing Pancreatic Cancer in the UK Biobank Cohort
Source: Cancer Med. 2025 Mar 25;14(6):e70809. doi: 10.1002/cam4.70809 (PMC11933721; doi:10.1002/cam4.70809)
Supplement: Supplementary file 1 — Data S1. [file CAM4-14-e70809-s001.docx]

Prospective associations of body composition and body shape with the risk of developing pancreatic cancer in the UK Biobank cohort

Sofia Christakoudi, Konstantinos K. Tsilidis, Marc J. Gunter, Elio Riboli

[S**upplementary Methods**………………………….……………………………………………………….2](#_Toc182479297)

**Supplementary Tables**

[Supplementary Table S1 Exclusion criteria 4](#_Toc182479298)

[Supplementary Table S2 Regression coefficients for anthropometric residuals 5](#_Toc182479299)

**Supplementary Figures**

[Supplementary Figure S1 Pairwise associations of covariates with exposures and pancreatic cancer risk 6](#_Toc182479300)

[Supplementary Figure S2 Associations of alternative body shape indices with pancreatic cancer risk – sensitivity analyses 9](#_Toc182479301)

[Supplementary Figure S3 Independent associations of anthropometric indices with pancreatic cancer risk in women and men 11](#_Toc182479302)

[Supplementary Figure S4 Independent associations of residual body composition with pancreatic cancer risk in women and men 12](#_Toc182479303)

[Supplementary Figure S5 Independent associations of anthropometric indices and diabetes with pancreatic cancer risk – sensitivity analyses 13](#_Toc182479304)

[Supplementary Figure S5 Independent associations of anthropometric indices and diabetes with pancreatic cancer risk – sensitivity analyses (continued) 14](#_Toc182479305)

[References………………………………………………………………………………………………….16](#_Toc182479306)

## Supplementary Methods

**Abdominal volume index (AVI)** is based on geometric considerations approximating body shape to a conus or a cylinder [17].

**Body roundness index** **(BRI)** is based on geometric considerations approximating body shape to an ellipse, with larger eccentricity (degree of circularity) in individuals with larger abdominal size [18]. Shown is the transformation of the equation to use waist-to-height ratio (WHtR) instead of waist circumference (WC) and height:

$$BRI=364.2-365.5*\sqrt{1-\frac{\left( \frac{0.5*WC\left( m \right)}{\pi} \right)^{2}}{\left( 0.5*Height\left( m \right) \right)^{2}}}=364.2-365.5*\sqrt{1-\frac{\left( 0.5 \right)^{2}*\left( \frac{1}{\pi} \right)^{2}*{WC\left( m \right)}^{2}}{{\left( 0.5 \right)^{2}*Height\left( m \right)}^{2}}}$$

$$BRI=364.2-365.5*\sqrt{1-\frac{1}{\pi^{2}}*\left( \frac{WC\left( m \right)}{Height\left( m \right)} \right)^{2}}=364.2-365.5*\sqrt{1-\frac{{WHtR}^{2}}{\pi^{2}}}$$

**Conicity index (ConI)** is based on geometric considerations approximating body shape to a cylinder or two cones with joined bases [19]. Shown is the transformation of the equation to use body mass index (BMI, kg/m^2^) instead of weight (kg):

$$ConI=\frac{WC(m)}{0.109*\sqrt{\frac{Weight}{Height(m)}}}=\frac{1}{0.109}*\frac{WC\left( m \right)}{\frac{{Weight}^{\frac{1}{2}}}{{Height\left( m \right)}^{\frac{1}{2}}}}=9.174*WC\left( m \right)*{Weight}^{- \frac{1}{2}}* {Height\left( m \right)}^{\frac{1}{2}}$$

$$ConI=9.174*WC\left( m \right)*{Weight}^{- \frac{1}{2}}*\frac{{Height\left( m \right)}^{- \frac{2}{2}}}{{Height\left( m \right)}^{- \frac{2}{2}}}*{Height\left( m \right)}^{\frac{1}{2}}$$

$$ConI=9.174*WC\left( m \right)*\frac{{Weight}^{- \frac{1}{2}}}{{Height\left( m \right)}^{- \frac{2}{2}}}*{{Height\left( m \right)}^{- \frac{2}{2}}*Height\left( m \right)}^{\frac{1}{2}}$$

$$ConI=9.174*WC\left( m \right)*\left( \frac{Weight}{{Height\left( m \right)}^{2}} \right)^{- \frac{1}{2}}*{Height\left( m \right)}^{\left( - \frac{2}{2} + \frac{1}{2} \right)}$$

$$ConI=9.174*WC(m)*{BMI}^{- \frac{1}{2}}*{Height\left( m \right)}^{- \frac{1}{2}}$$

**Weight-adjusted waist index (WWI)** is an allometric index scaling WC for weight in a Korean population [20]. Shown is the transformation of the equation to use BMI (kg/m^2^) instead of weight (kg), including a conversion of the unit of measurement of WC from (cm) to (m):

$$WWI=WC\left( cm \right)*{Weight}^{- \frac{1}{2}}=100*WC (m)*{Weight}^{- \frac{1}{2}}*\frac{{Height(m)}^{- \frac{2}{2}}}{{Height(m)}^{- \frac{2}{2}}}$$

$$WWI=100*WC \left( m \right)*\frac{{Weight}^{- \frac{1}{2}}}{{Height(m)}^{- \frac{2}{2}}}*{Height\left( m \right)}^{- \frac{2}{2}}$$

$$WWI=100*WC \left( m \right)*\left( \frac{Weight}{{Height(m)}^{2}} \right)^{- \frac{1}{2}}*{Height\left( m \right)}^{-1}$$

$$WWI=100*WC\left( m \right)*{BMI}^{- \frac{1}{2}}*{Height(m)}^{-1}$$

**Waist-to-hip index (WHI)** is an allometric index scaling the waist-to-hip ratio (WHR) for weight and height previously developed by us in UK Biobank [8]. Shown is the transformation of the equation to use BMI (kg/m^2^) instead of weight (kg) and height, including a conversion of the unit of measurement of height from (cm) to (m):

$$WHI=WHR*{Weight}^{- \frac{1}{4}}*{Height\left( cm \right)}^{\frac{1}{2}}=WHR*{Weight}^{- \frac{1}{4}}*\left( 100*Height\left( m \right) \right)^{\frac{1}{2}}$$

$$WHI=WHR*{Weight}^{- \frac{1}{4}}*\frac{{Height(m)}^{- \frac{2}{4}}}{{Height(m)}^{- \frac{2}{4}}}*{100}^{\frac{1}{2}}*{Height\left( m \right)}^{\frac{1}{2}}$$

$$WHI=WHR*\frac{{Weight}^{- \frac{1}{4}}}{{Height(m)}^{- \frac{2}{4}}}*{Height(m)}^{- \frac{1}{2}}*{10*Height\left( m \right)}^{\frac{1}{2}}$$

$$WHI=10*WHR*\left( \frac{Weight}{{Height(m)}^{2}} \right)^{- \frac{1}{4}}*{Height\left( m \right)}^{\left( - \frac{1}{2} + \frac{1}{2} \right)}$$

$$WHI=10* WHR*{BMI}^{- \frac{1}{4}}*{Height\left( m \right)}^{0}=10* WHR*{BMI}^{- \frac{1}{4}}$$

**A body shape index (ABSI)** is an allometric index scaling WC for weight and height developed in a North American population [21]. Shown is the transformation of the equation to use BMI (kg/m^2^) instead of weight (kg):

$$ABSI=1000*WC\left( m \right)*{Weight}^{- \frac{2}{3}}*{Height\left( m \right)}^{\frac{5}{6}}$$

$$ABSI=1000*WC\left( m \right)*{Weight}^{- \frac{2}{3}}*\frac{{Height\left( m \right)}^{- \frac{4}{3}}}{{Height\left( m \right)}^{- \frac{4}{3}}}*{Height\left( m \right)}^{\frac{5}{6}}$$

$$ABSI=1000*WC\left( m \right)*\frac{{Weight}^{- \frac{2}{3}}}{{Height\left( m \right)}^{- \frac{4}{3}}}*{{Height\left( m \right)}^{- \frac{4}{3}}*Height\left( m \right)}^{\frac{5}{6}}$$

$$ABSI=1000*WC(m)*\left( \frac{Weight}{{Height(m)}^{2}} \right)^{- \frac{2}{3}}*{Height(m)}^{\left( - \frac{8}{6} + \frac{5}{6} \right)}$$

$$ABSI=1000*WC(m)*{BMI}^{- \frac{2}{3}}*{Height(m)}^{- \frac{1}{2}}$$

## Supplementary Table S1 Exclusion criteria

|  |  | Total | Women | Men |
| --- | --- | --- | --- | --- |
|  | Total available | 502,172 | 273,182 | 228,990 |
| 1. | Ethnic background (restricted to self-reported white) | 29,772 | 15,919 | 13,853 |
| 2. | Anthropometric measurements missing | 2265 | 1123 | 1142 |
| 3. | Bioelectrical impedance measurements missing | 7602 | 3517 | 4085 |
| 4. | Genetic & self-reported sex mismatch, or sex chromosome  aneuploidy, or pregnant at recruitment | 776 | 386 | 390 |
| 5. | Prevalent cancer at recruitment | 33,818 | 21,833 | 11,985 |
|  | Total excluded: | 74,233 | 42,778 | 31,455 |
|  | (% from the available dataset) | (14.8) | (15.7) | (13.7) |
|  | Total included: | 427,939 | 230,404 | 197,535 |

The exclusion criteria were applied sequentially in the displayed order, counting each excluded individual only once. Participants withdrawing consent by the time of analysis were excluded from the total available. For UK Biobank field names, definition of variables, and definition of prevalent cancer cases see Supplementary Methods in reference [8].

## Supplementary Table S2 Regression coefficients for anthropometric residuals

| **Residual FM** | Intercept | Height |  | R^2^ |
| --- | --- | --- | --- | --- |
| Women | 0 (0.0207) | 0.2019 (0.0033) |  | 0.016 |
| Men | 0 (0.0184) | 0.1316 (0.0027) |  | 0.012 |
| **Residual FFM** | Intercept | Height ^#^ | Total FM | R^2^ |
| Women | 0 (0.0062) | 0.3198 (0.0010) | 0.3182 (0.0006) | 0.638 |
| Men | 0 (0.0102) | 0.6461 (0.0015) | 0.4832 (0.0013) | 0.651 |
| **WHRadjBMI** | Intercept |  | BMI | R^2^ |
| Women | 0 (0.00013) |  | 0.00627 (0.00003) | 0.214 |
| Men | 0 (0.00012) |  | 0.00921 (0.00003) | 0.358 |

**BMI** – body mass index; **FM** – fat mass (bioelectrical impedance); **FFM** – fat-free mass (bioelectrical impedance); **R^2^** – proportion explained variance; **WHR** – waist-to-hip ratio; **WHRadjBMI** – waist-to-hip ratio adjusted for BMI (residuals of WHR regressed on BMI).

Regression coefficients (standard error) were derived from the following models, after centring all variables with the sex-specific mean:

$$Total FM \left( kg \right) \sim Height \left( cm \right)$$

$$Total FFM \left( kg \right) \sim Height (cm)+Total FM \left( kg \right)$$

$$WHR \sim BMI \left( {kg/m}^{2} \right)$$

**^#^** Height alone explained 0.233 of the variance of total FFM in women and 0.387 in men.


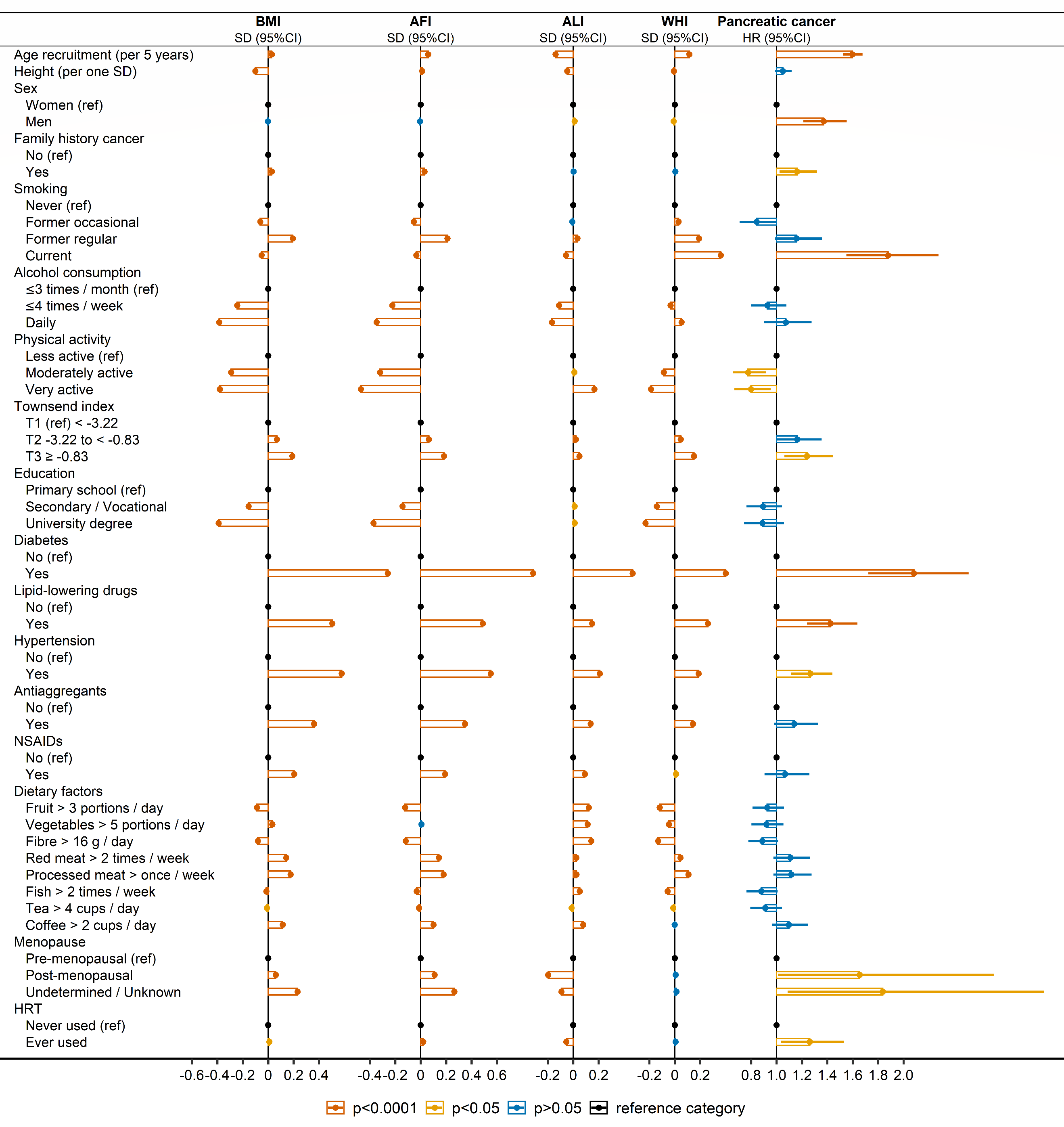


## Supplementary Figure S1 Pairwise associations of covariates with exposures and pancreatic cancer risk

**AFI** – allometric fat-mass index; **ALI** – allometric lean-mass index; **BMI** – body mass index; **CI** – confidence interval; **HR** – hazard ratio; **HRT** – hormone replacement therapy; **NSAID** – non-steroidal anti-inflammatory drugs; **SD** – standard deviation; **T1-T3** – cohort-specific tertiles; **WHI** – waist-to-hip index (allometric).

Estimates from liner regression models (SD scale) including individually each anthropometric index specified in the header as outcome and adjusted for sex and age at recruitment (continuous) or from Cox proportional hazards models (HR) with timescale age at recruitment (or person years of follow-up when examining age at recruitment as exposure), stratified by sex. Each covariate was included individually as the independent variable. Associations with menopausal status were examined in women. Associations with HRT were examined in the combined group of women post-menopausal or with undetermined/unknown menopause at recruitment, as very few pre-menopausal women had used HRT.

**Definition of covariates**

The following covariates were defined as previously described in reference [8]: family history of cancer (lung, bowel, prostate, or breast cancer in parents or siblings), smoking status, alcohol consumption, physical activity, and Townsend deprivation index (cohort-specific instead of sex-specific tertiles).

Education was based on Fields [6138-0.0/5] “*Qualifications*”, question “*Which of the following qualifications do you have? (You can select more than one)*”. Categories were defined as follows: Answer 1 “*College or University degree*” for category “University degree”; else any of Answers 2 “*A levels/AS levels or equivalent*”, 3 “*O levels/GCSEs or equivalent*”, 4 “*CSEs or equivalent*”, 5 “*NVQ or HND or HNC or equivalent*”, 6 “*Other professional qualifications eg: nursing, teaching*” for category “Secondary / Vocational”; else Answer -7 “*None of the above*” for category “Primary school”; and considering Answer -3 “*Prefer not to answer*” as missing information.

Diabetes-Yes included participants with self-reported diabetes or using anti-diabetic drugs (defined as in reference [66]), or with glycated haemoglobin ≥48 mmol/mol from Field [30750-0.0]. Lipid-lowering drugs were defined as in reference [66] and additionally included cholestyramine products. Use of NSAIDs and antiaggregant/anticoagulants was defined according to reference [67].

Hypertension was based on Fields [6150-0.0/3] “*Vascular/heart problems diagnosed by doctor*”, Question: “*Has a doctor ever told you that you have had any of the following conditions? (You can select more than one answer)*”, Answer: 4 “*High blood pressure*” for category Yes; else any of Answers -7 “*None of the above*”, 1 “*Heart attack*”, 2 “*Angina*”, or 3 “*Stroke*” for category No; and considering Answer -3 “*Prefer not to answer*” as missing information. We further re-classified to category Yes participants with self-reported hypertension with codes: 1065 “*hypertension*” or 1072 “*essential hypertension*” in Fields [20002-0.0/33] “*Non-cancer illness code, self-reported*” and included in category Yes all participants using antihypertensive drugs defined as in reference [66].

Fruit intake was based on the sum of Field [1309-0.0] *“Fresh fruit intake”* and Field [1319-0.0] *“Dried fruit intake”.* Vegetable intake was based on the sum of Field [1299-0.0] *“Salad / raw vegetable intake”* and Field [1289-0.0] *“Salad / raw vegetable intake”.* Tea intake was based on Field [1488-0.0] *“Tea intake”.* Coffee intake was based on Field [1498-0.0] *“Coffee intake”.* Answer -10 *"Less than one"* was re-coded to 0.5. Answers -1 *“Do not know”* and -3 *“Prefer not to answer”* were considered missing values. Fibre intake was defined as the sum of fibre obtained from fruit, vegetables, bread, and cereals, as described in reference [12].

Red meat intake was based on the sum of Field [1369-0.0] *“Beef intake”*, Field [1379-0.0] *“Lamb/mutton intake”*, and Field [1389-0.0] *“Pork intake”*. Processed meat intake was based on Field [1349-0.0] *“Processed meat intake”*. Fish intake was based on the sum of Field [1329-0.0] *“Oily fish intake”* and Field [1339-0.0*] “Non-oily fish intake”*. Categorical answers for meat variables were converted to a continuous scale as described in reference [12]: 0 for Answer 0 *“Never”;* 0.5 for Answer 1 *“Less than once a week”*; 1 for Answer 2 *“Once a week”*; 3 for Answer 3 *“2-4 times a week”*; 5.5 for Answer 4 *“5-6 times a week”*; 7 for Answer 5 *“Once or more daily”*. Answers -1 *“Do not know”* and -3 *“Prefer not to answer”* were considered missing values.

All dietary variables were dichotomised with respect to the upper cohort-specific tertile boundary.

Menopausal status and HRT use were defined similarly to reference [68], combining for this study current and past users as ever HRT users.

**Missing values**

Smoking status 1376 (0.3% of study cohort); alcohol consumption 300 (0.1); physical activity 1459 (0.3); education 6862 (1.6); Townsend deprivation index 501 (0.1); diabetes 855 (0.2); lipid-lowering drugs 5130 (1.2); hypertension 624 (0.1); NSAIDs 6911 (1.6); antiaggregant/anticoagulants 6836 (1.6); fruit 661 (0.2); vegetables 2445 (0.6); fibre 51 (<0.1); red meat 575 (0.1); processed meat 610 (0.1); fish 549 (0.1); tea 823 (0.2); coffee 687 (0.2); HRT 400 (0.2% of women post-menopausal or with undetermined/unknown menopause, n=174,899).

Missing values were replaced with the sex-specific median category.

There were 25,726 women with undetermined/unknown menopause (11.2% of all women), which were considered jointly with 149,173 post-menopausal women (64.7% of all women).

**Associations of covariates with pancreatic cancer risk**

Higher pancreatic cancer risk has been reported for smoking and alcohol consumption [69]; red and processed meat consumption [70]; sedentary behaviour [71]; metabolic syndrome overall and its individual components [72]; coffee consumption [73]. Lower pancreatic cancer risk has been reported for fruit, vegetable, and whole grain consumption [70]; dietary fibre [74]; physical activity [75]. Reports for associations of HRT with pancreatic cancer risk are inconsistent, but some studies have found lower risk for oestrogen only or combined preparations [76]. Lower pancreatic cancer risk has been reported for use of aspirin but not for use of NSAIDs [77], although the risk of pancreatic cancer was higher in individuals with high levels of biomarkers of chronic inflammation [78]. Little evidence for association of tea consumption with pancreatic cancer risk has been found [79] but based on smaller studies with no recent meta-analysis.





## Supplementary Figure S2 Associations of alternative body shape indices with pancreatic cancer risk – sensitivity analyses

**ABSI** – a body shape index; **AVI** – abdominal volume index; **BMI** – body mass index; **BRI** – body roundness index; **CI** – confidence interval; **ConI** – conicity index; **HC** – hip circumference; **HI** – hip index; **HR** – hazard ratio; **SD** – standard deviation; **WC** – waist circumference; **WHI** – waist-to-hip index; **WHR** – waist-to-hip ratio; **WHRadjBMI** – waist-to-hip ratio adjusted for BMI (residuals of WHR regressed on BMI); **WHtR** – waist-to-height ratio; **correlation** – pairwise partial Pearson correlation coefficient adjusted for sex; **p-value** – Wald test for the individual term; **difference** – between the HR estimates of adjusted models compared to the unadjusted model (stratified only by sex).

**Sex** – estimates from unadjusted Cox proportional hazards models in participants overall (stratified only by sex), including individually as exposure variable one of the listed body shape measures or indices (sex-specific z-scores, value minus mean divided by SD). BMI is shown for comparison.

**Sex & Smoking** – models stratified by sex and adjusted for smoking status.

**Sex** **& Smoking & Diabetes** – models stratified by sex and adjusted for smoking status and diabetes.

**Fully adjusted** – models stratified by age and a combined variable of sex, menopausal status, and hormone replacement therapy use, and adjusted for smoking status, alcohol consumption, physical activity, education, Townsend deprivation index, family history of cancer, diabetes, hypertension, use of lipid-lowering drugs, non-steroidal anti-inflammatory drugs, and antiaggregant/anticoagulants, and dietary intake of fruit, vegetables, fibre, red meat, processed meat, fish, tea and coffee.


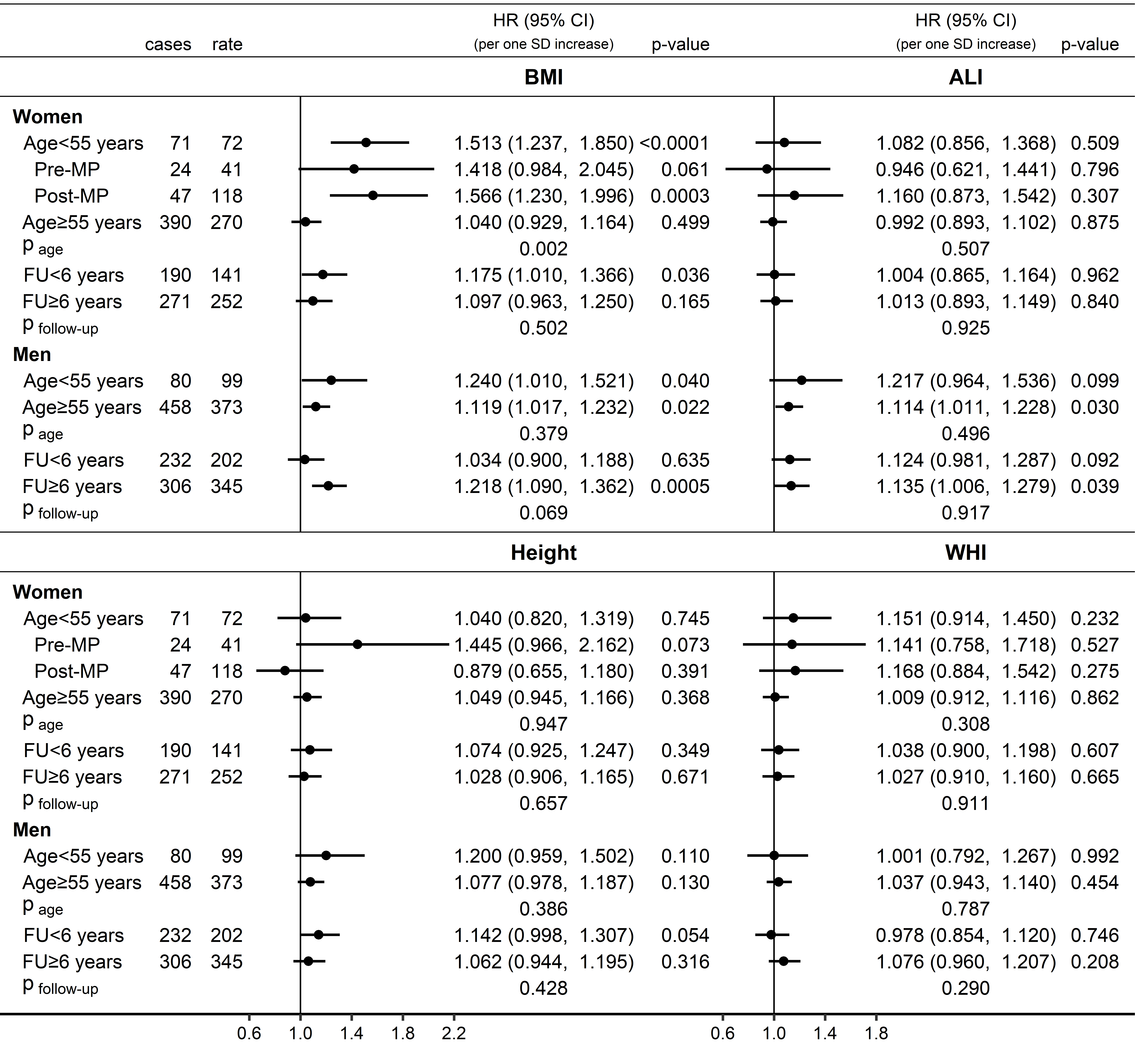


### Supplementary Figure S3 Independent associations of anthropometric indices with pancreatic cancer risk in women and men

**ALI** – allometric lean-mass index; **BMI** – body mass index; **CI** – confidence interval; **FU** – follow-up time; **HR** – hazard ratio; **Pre-MP** – women pre-menopausal at recruitment; **Post-MP** – women post-menopausal at recruitment or with undetermined/unknown menopausal status; **cases** – number of pancreatic cancer cases per group; **rate** – incidence rate per 1*10^6^ person years; **p-value** – Wald test for the individual term; **SD** – standard deviation; **WHI** – waist-to-hip index.

Estimates from multivariable Cox proportional hazards models including jointly as exposure variables allometric fat-mass index, ALI, WHI, height (sex-specific z-scores, value minus mean divided by SD) and diabetes, stratified by age and a combined variable of sex, menopausal status, and hormone replacement therapy use, and adjusted for smoking status, alcohol consumption, physical activity, education, Townsend deprivation index, family history of cancer, hypertension, use of lipid-lowering drugs, non-steroidal anti-inflammatory drugs, and antiaggregant/anticoagulants, and dietary intake of fruit, vegetables, fibre, red meat, processed meat, fish, tea, and coffee (same as in Figure 5 in the main document). Estimates for BMI were obtained from similar joint fully adjusted models including BMI instead of allometric fat-mass index and ALI.

**p _age_ / p _follow-up_** – p-value obtained with the data augmentation method of Lunn and McNeil [24] for the comparison of HR estimates between the specified groups according to age at recruitment (<55 years, ≥55 years) and follow-up time (<6 years, ≥6 years).


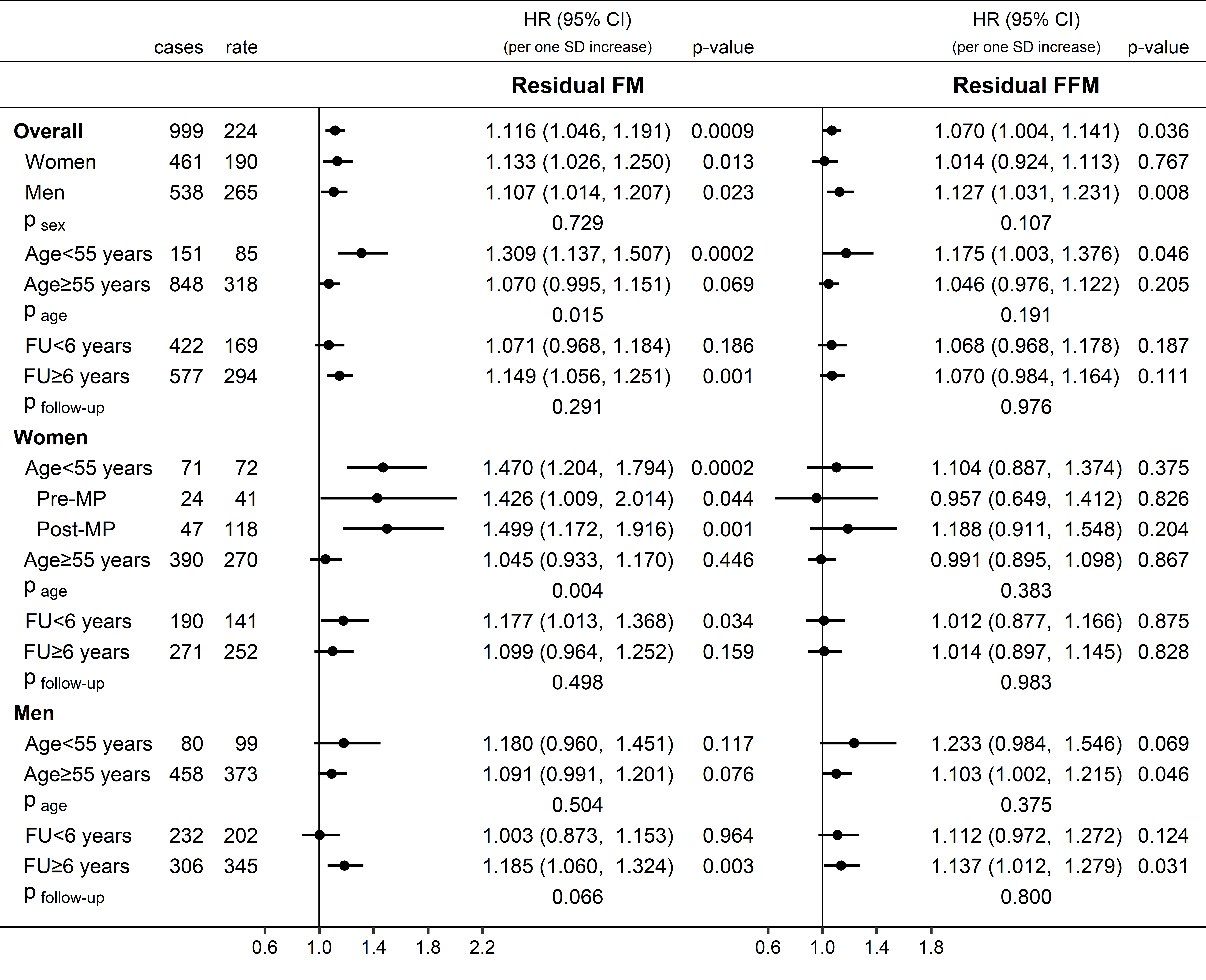


### Supplementary Figure S4 Independent associations of residual body composition with pancreatic cancer risk in women and men

**CI** – confidence interval; **FM** – fat mass; **FFM** – fat-free mass; **FU** – follow-up time; **HR** – hazard ratio; **Pre-MP** – women pre-menopausal at recruitment; **Post-MP** – women post-menopausal at recruitment or with undetermined/unknown menopausal status; **Residual FM** & **FFM** – residuals of total FM (kg) regressed on height (cm) or total FFM (kg) regressed on height (cm) and total FM (kg); **SD** – standard deviation; **cases** – number of pancreatic cancer cases per group; **rate** – incidence rate per 1*10^6^ person years in each group; **p-value** – Wald test for the individual term.

Estimates from multivariable Cox proportional hazards models including jointly as exposure variables residual FM, residual FFM, waist-to-hip index, height (sex-specific z-scores, value minus mean divided by SD) and diabetes, stratified by age and a combined variable of sex, menopausal status, and hormone replacement therapy use, and adjusted for smoking status, alcohol consumption, physical activity, education, Townsend deprivation index, family history of cancer, hypertension, use of lipid-lowering drugs, non-steroidal anti-inflammatory drugs, and antiaggregant/anticoagulants, and dietary intake of fruit, vegetables, fibre, red meat, processed meat, fish, tea, and coffee.

**p _sex_ / p _age_ / p _follow-up_** – p-value obtained with the data augmentation method of Lunn and McNeil [24] for the comparison of HR estimates between the specified groups according to sex (women, men), age at recruitment (<55 years, ≥55 years), and follow-up time (<6 years, ≥6 years).

**

**

## Supplementary Figure S5 Independent associations of anthropometric indices and diabetes with pancreatic cancer risk – sensitivity analyses


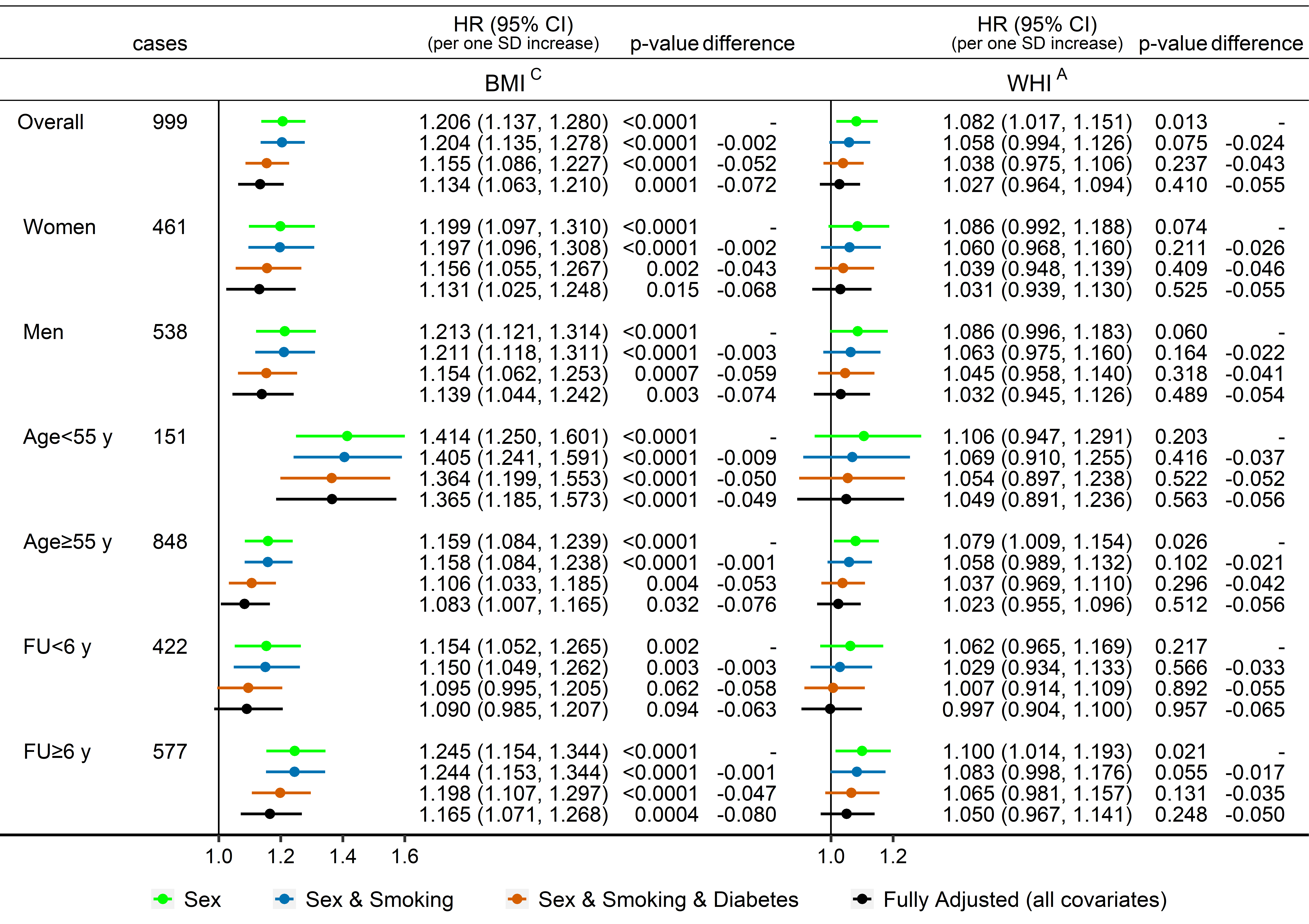


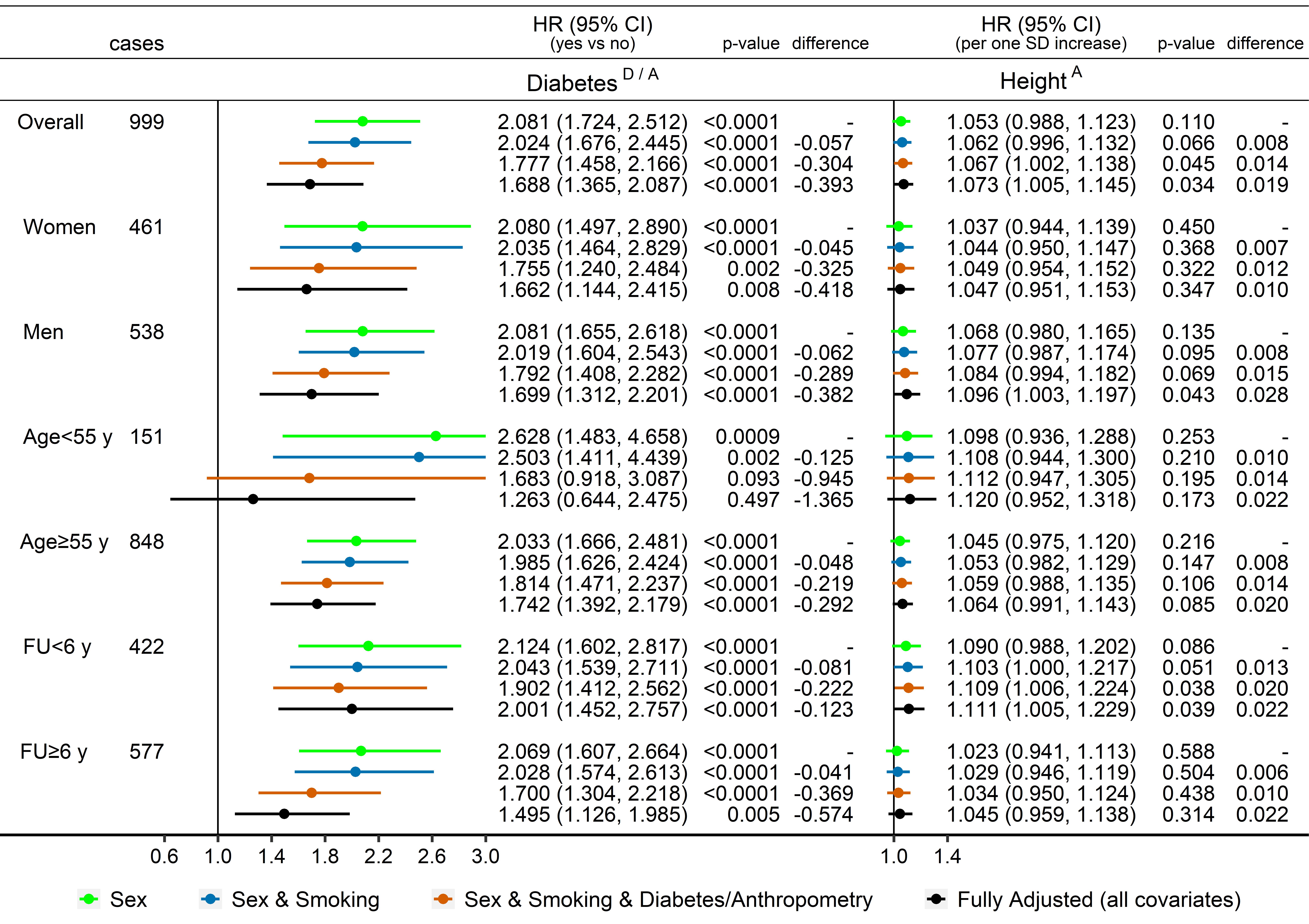


## Supplementary Figure S5 Independent associations of anthropometric indices and diabetes with pancreatic cancer risk – sensitivity analyses (continued)

**AFI** – allometric fat-mass index; **ALI** – allometric lean-mass index; **BMI** – body mass index; **CI** – confidence interval; **FM** – fat mass; **FFM** – fat-free mass; **FU** – follow-up time; **HR** – hazard ratio; **SD** – standard deviation; **WHI** – waist-to-hip index; **Residual FM** – residuals of total FM (kg) regressed on height (cm); **Residual FFM** – residuals of total FFM (kg) regressed on height (cm) and total FM (kg); **cases** – number of pancreatic cancer cases per group; **p-value** – Wald test for the individual term.

**Sex** – estimates from unadjusted Cox proportional hazards models in participants overall (stratified only by sex), including jointly as exposure variables AFI, ALI, WHI and height (model A), or residual FM, residual FFM, WHI and height (model B), or BMI, WHI and height (model C) (sex-specific z-scores, value minus mean divided by SD), or individually diabetes (model D).

**Sex & Smoking** – models A, B, C, or D as described above, adjusted for smoking status.

**Sex** **& Smoking & Diabetes/Anthropometry** – models A, B, or C with added diabetes, stratified by sex, and adjusted for smoking status.

**Fully adjusted** – models A, B, or C with added diabetes, stratified by age and a combined variable of sex, menopausal status, and hormone replacement therapy use, and adjusted for smoking status, alcohol consumption, physical activity, education, Townsend deprivation index, family history of cancer, hypertension, use of lipid-lowering drugs, non-steroidal anti-inflammatory drugs, and antiaggregant/anticoagulants, and dietary intake of fruit, vegetables, fibre, red meat, processed meat, fish, tea, and coffee.

References

**References cited in the main document**:

8. Christakoudi S, Tsilidis KK, Evangelou E, Riboli E. A Body Shape Index (ABSI), hip index and risk of cancer in the UK Biobank cohort. *Cancer Med*. 2021;10(16):5614-28. <https://doi.org/10.1002/CAM4.4097>

12. Christakoudi S, Tsilidis KK, Evangelou E, Riboli E. Association of body-shape phenotypes with imaging measures of body composition in the UK Biobank cohort: relevance to colon cancer risk. *BMC cancer*. 2021;21(1):1106. <https://doi.org/10.1186/s12885-021-08820-6>

17. Guerrero-Romero F, Rodriguez-Moran M. Abdominal volume index. An anthropometry-based index for estimation of obesity is strongly related to impaired glucose tolerance and type 2 diabetes mellitus. *Arch Med Res*. 2003;34(5):428-32. <https://doi.org/10.1016/s0188-4409(03)00073-0>

18. Thomas DM, Bredlau C, Bosy-Westphal A, Mueller M, Shen W, Gallagher D et al. Relationships between body roundness with body fat and visceral adipose tissue emerging from a new geometrical model. *Obesity (Silver Spring, Md)*. 2013;21(11):2264-71. <https://doi.org/10.1002/oby.20408>

19. Valdez R, Seidell JC, Ahn YI, Weiss KM. A new index of abdominal adiposity as an indicator of risk for cardiovascular disease. A cross-population study. *Int J Obes Relat Metab Disord*. 1993;17(2):77-82.

20. Park Y, Kim NH, Kwon TY, Kim SG. A novel adiposity index as an integrated predictor of cardiometabolic disease morbidity and mortality. *Sci Rep*. 2018;8(1):16753. <https://doi.org/10.1038/s41598-018-35073-4>

21. Krakauer NY, Krakauer JC. A New Body Shape Index Predicts Mortality Hazard Independently of Body Mass Index. *PLoS One*. 2012;7(7):e39504. <https://doi.org/10.1371/journal.pone.0039504>

24. Lunn M, McNeil D. Applying Cox regression to competing risks. *Biometrics*. 1995;51(2):524-32.

**Supplementary references**:

66. Christakoudi S, Riboli E, Evangelou E, Tsilidis KK. Associations of body shape index (ABSI) and hip index with liver, metabolic, and inflammatory biomarkers in the UK Biobank cohort. Sci Rep. 2022;12(1):8812. <https://doi.org/10.1038/s41598-022-12284-4>

67. Christakoudi S, Tsilidis KK, Evangelou E, Riboli E. Interactions of platelets with obesity in relation to lung cancer risk in the UK Biobank cohort. *Respir Res*. 2023;24(1):249. <https://doi.org/10.1186/s12931-023-02561-9>

68. Christakoudi S, Riboli E, Evangelou E, Tsilidis KK. Associations of body shape phenotypes with sex steroids and their binding proteins in the UK Biobank cohort. *Sci Rep*. 2022;12(1):10774. <https://doi.org/10.1038/s41598-022-14439-9>

69. Korc M, Jeon CY, Edderkaoui M, Pandol SJ, Petrov MS. Tobacco and alcohol as risk factors for pancreatic cancer. *Best Pract Res Clin Gastroenterol*. 2017;31(5):529-36. <https://doi.org/10.1016/j.bpg.2017.09.001>

70. Gianfredi V, Ferrara P, Dinu M, Nardi M, Nucci D. Diets, Dietary Patterns, Single Foods and Pancreatic Cancer Risk: An Umbrella Review of Meta-Analyses. *Int J Environ Res Public Health*. 2022;19(22). <https://doi.org/10.3390/ijerph192214787>

71. Gentiluomo M, Dixon-Suen SC, Farinella R, Peduzzi G, Canzian F, Milne RL et al. Physical Activity, Sedentary Behavior, and Pancreatic Cancer Risk: A Mendelian Randomization Study. *J Endocr Soc*. 2024;8(4):bvae017. <https://doi.org/10.1210/jendso/bvae017>

72. Zhong L, Liu J, Liu S, Tan G. Correlation between pancreatic cancer and metabolic syndrome: A systematic review and meta-analysis. *Front Endocrinol (Lausanne)*. 2023;14:1116582. <https://doi.org/10.3389/fendo.2023.1116582>

73. Li TD, Yang HW, Wang P, Song CH, Wang KJ, Dai LP et al. Coffee consumption and risk of pancreatic cancer: a systematic review and dose-response meta-analysis. *Int J Food Sci Nutr*. 2019;70(5):519-29. <https://doi.org/10.1080/09637486.2018.1551337>

74. Nucci D, Santangelo OE, Provenzano S, Fatigoni C, Nardi M, Ferrara P et al. Dietary Fiber Intake and Risk of Pancreatic Cancer: Systematic Review and Meta-Analysis of Observational Studies. *Int J Environ Res Public Health*. 2021;18(21). <https://doi.org/10.3390/ijerph182111556>

75. Xie F, You Y, Huang J, Guan C, Chen Z, Fang M et al. Association between physical activity and digestive-system cancer: An updated systematic review and meta-analysis. *J Sport Health Sci*. 2021;10(1):4-13. <https://doi.org/10.1016/j.jshs.2020.09.009>

76. Jang YC, Leung CY, Huang HL. Association of Menopausal Hormone Therapy with Risk of Pancreatic Cancer: A Systematic Review and Meta-analysis of Cohort Studies. *Cancer Epidemiol Biomarkers Prev*. 2023;32(1):114-22. <https://doi.org/10.1158/1055-9965.Epi-22-0518>

77. Zhang YP, Wan YD, Sun YL, Li J, Zhu RT. Aspirin might reduce the incidence of pancreatic cancer: A meta-analysis of observational studies. *Sci Rep*. 2015;5:15460. <https://doi.org/10.1038/srep15460>

78. Sollie S, Michaud DS, Sarker D, Karagiannis SN, Josephs DH, Hammar N et al. Chronic inflammation markers are associated with risk of pancreatic cancer in the Swedish AMORIS cohort study. *BMC Cancer*. 2019;19(1):858. <https://doi.org/10.1186/s12885-019-6082-6>

79. Chang B, Sang L, Wang Y, Tong J, Wang BY. Consumption of tea and risk for pancreatic cancer: a meta-analysis of published epidemiological studies. *Nutr Cancer*. 2014;66(7):1109-23. <https://doi.org/10.1080/01635581.2014.951730>
